# Metabolomics reveals an entanglement of fasting leptin concentrations with fatty acid oxidation and gluconeogenesis in healthy children

**DOI:** 10.1371/journal.pone.0183185

**Published:** 2017-08-17

**Authors:** Franca Fabiana Kirchberg, Stephanie Brandt, Anja Moß, Wolfgang Peissner, Wolfgang Koenig, Dietrich Rothenbacher, Hermann Brenner, Berthold Koletzko, Christian Hellmuth, Martin Wabitsch

**Affiliations:** 1 Ludwig-Maximilians-Universität München, Division of Metabolic and Nutritional Medicine, Dr. von Hauner Children’s Hospital, University of Munich Medical Center, Munich, Germany; 2 Department of Pediatrics and Adolescent Medicine, Division of Pediatric Endocrinology and Diabetes, University Medical Center Ulm, Ulm, Germany; 3 Department of Internal Medicine II-Cardiology, University of Ulm Medical Center, Ulm, Germany; 4 Deutsches Herzzentrum München, Technische Universität München, Munich, Germany; 5 DZHK (German Centre for Cardiovascular Research), partner site Munich Heart Alliance, Munich, Germany; 6 Institute of Epidemiology and Medical Biometry, University of Ulm, Ulm, Germany; 7 German Cancer Research Center (DKFZ), Division of Clinical Epidemiology and Aging Research, Heidelberg, Germany; Shanghai Jiaotong University School of Medicine Xinhua Hospital, CHINA

## Abstract

**Background:**

Leptin and adiponectin communicate with organ systems in order to regulate energetic and metabolic homeostasis. Their different points of action have been well characterized; however, no study has investigated their interrelationship with the metabolism at the molecular level *in vivo*.

**Objective:**

To examine the associations of leptin and adiponectin with the metabolic profile reflecting the intercellular and interorgan communication as well as activated metabolic pathways.

**Patients/Methods:**

We measured plasma concentrations of leptin, adiponectin, and insulin along with concentrations of 196 metabolites in 400 healthy, fasting 8-years old German children who participated in the German Ulm Birth Cohort Study (UBCS). Using multiple linear mixed models, we evaluated the associations between hormones and metabolites.

**Results:**

Leptin levels increased exponentially with increasing BMI. Leptin was furthermore strongly associated with alanine and aspartate (Bonferroni corrected *P*[*P*_BF_] = 5.7×10^−8^ and 1.7×10^−6^, respectively), and negatively associated to the sum of the non-esterified fatty acids (NEFA) and the sum of the long-chain acylcarnitines C12–C18 (*P*_BF_ = 0.009 and 0.0001, respectively). Insulin showed a similar association pattern, although the associations were less strong than for leptin. Adiponectin was neither related to BMI nor to any metabolite.

**Conclusion:**

Although children were presumably metabolically similar, we found strong associations of insulin and leptin with the metabolite profile. High alanine concentrations and the lower concentrations of NEFA in children with high fasting leptin concentrations might arise from an increased gluconeogenesis and from the disinhibiting effect of leptin on the carnitine-palmitoyltransferase-1, respectively. As insulin had the same trend towards these associations, both hormones seem to be related to processes that provide the body with energy in fasting state.

## Introduction

While many mechanisms of action of the adipokines leptin and adiponectin, such as fatty acid oxidation enhancing the effect of leptin or the insulin sensitizing effect of adiponectin, have been elucidated, some other effects, like their role in glucose metabolism, are not yet fully understood because different studies reported controversial results [1, 2]. Furthermore, many of these studies are done on mice models and it remains to be clarified what these animal models reveal about humans [3]. Another unsolved mystery is that of leptin resistance. Leptin, despite being secreted by adipocytes and exerting anorexic effects, is elevated in obese compared to lean subjects [4]. This antagonism is explained by the leptin resistance that develops in adipose individuals [5]. The need for studying the adipokine effects in humans is further highlighted by the involvement of the adipokines and other hormones in early programming pathways. During fetal and early life, the metabolism of humans is more plastic compared to later periods and environmental factors, such as diet, or stress exposures were shown to influence later health [6]. Leptin and adiponectin were found to be involved in (re-)programming pathways affecting later health [7]. Hence, there is a need for a more comprehensive and complete picture on the associations of these adipokines with the human metabolism in childhood. Metabolomics, the study of small-molecule metabolite profiles, is a widely used tool to characterize molecular mechanisms in organisms [8]. The metabolites are intermediates and end products of cellular regulatory processes and their levels can be regarded as the result of the interaction of genome, transcriptome, proteome, and the environment [9].

As part of the prospective Ulm Birth Cohort Study (UBCS), we measured 196 metabolites in plasma of 8 years old healthy children. We related the adipokine concentrations to the concentration of the metabolites and thus provide an insight into the involvements of the adipokines in energy homeostasis pathways in healthy children where the confounding effect of leptin resistance should be minimized.

## Subjects and methods

### Study design and population

The UBCS is a prospective birth cohort study. Women who gave birth at the Department of Gynaecology and Obstetrics at the University of Ulm between November 2000 and November 2001 were recruited. A total of 1593 mother-newborn pairs fulfilled the following inclusion criteria: understanding of German, Turkish, or Russian language, pregnancy duration of at least 32 weeks, birth weight >2000g, infants remaining in the basic care nursery (level 1) and the mothers staying at postnatal ward of the Department of Gynecology and Obstetrics after delivery. Of these, 1066 agreed to participate in the study. There have been regular follow-up examinations including age 8 years, when fasted blood samples were obtained from the children. For more information see [10].

Participation was voluntary and informed consent was obtained in each case. The study was approved by the ethics boards of the Universities of Ulm and Heidelberg and of the physicians' boards of the states of Baden-Wuerttemberg and Bavaria.

### Sample collection

At 8 years follow-up, plasma samples of the children were obtained after an overnight fast of at least 10 h between 8 and 9 a.m. (n = 486). Out of these, 400 children had adiponectin or leptin measurement along with metabolites measurements (29 had neither measurement, two had metabolite but no adipokine measurement, 55 had adipokine but no metabolite measurement). Blood samples were processed and plasma aliquots were stored at -80°C.

### Determination of hormones and glucose

The fasting plasma concentrations of leptin (μg/l),total adiponectin (μg/ml) and insulin (mU/l) were measured in a batch in October 2010 using a commercially available ELISA (Mercodia, leptin: coefficient of variation [CV] 7.00%, adiponectin: 6.47%, insulin: 5.06%). Concentrations of fasting plasma glucose were measured by using HemoCue B-Glucose Analyzer (Quest Diagnostics, Spain). HOMA was calculated: insulin (mU/l) x glucose (mg/dl)/405. All laboratory measures were performed in a blinded fashion.

### Determination of metabolites

Measurements of plasma metabolites were performed at the laboratory of the Division of Metabolic and Nutritional Medicine, Dr. von Hauner Children’s Hospital, Ludwig-Maximilians-Universität München. Amino acids (AA) were determined by ion-pair liquid chromatography. Polar lipids (acylcarnitines, diacyl-phosphatidylcholines [PCaa], acyl-alkyl-phosphatidylcholines [PCae], sphingomyelines [SM], acyl-[LPCa], alkyl-lysophosphatidylcholines [LPCe] and sum of hexoses) were analyzed with flow-injection analysis-tandem mass spectrometry. Non-esterified acids (NEFA) were quantified by LC-MS/ MS analysis. For detailed information on all measurement methods see [11].

NEFA and polar lipids are mentioned as CX:Y (X denomiates the length of the carbon chain, Y the number of double bonds). OH in the formula indicates that the molecule contains a hydroxyl-group. “a” indicates that the acyl chain is bound via an ester bond to the backbone; “e” indicates binding by an ether bound. We report all metabolite concentrations in μmol/L.

We furthermore calculated the sum of all acylcarnitines that mainly use the carnitine shuttle to enter the mitochondrion (C12:1, C14:1, C16:0, C18:0, C18:1 [12, 13]), the acylcarnitine ratios (C16+C18)/C0 and C2/(C16+C18) as marker for CPT-1 and fatty acid oxidation activity, respectively, as well as the sum of all NEFA. Taken together, we analyzed 200 metabolites and metabolites ratios/sums.

### Statistical analysis

To quantify measurement accuracy, six plasma quality control (QC) samples were consistently measured twice along with the samples per batch. We calculated the coefficient of variation (CV) for each QC sample across the batches and excluded metabolites whose CV was >35%.

We screened the metabolite concentrations graphically and by using principal component analysis and excluded outliers defined as observations that lay over 1 SD away from its next neighbor from the analyses. The aim of the principal analysis was to capture the association between the adipokine levels, namely leptin and adiponectin, and the plasma metabolic profile. Associations between the adipokines and standard deviation score of body mass index (SDS-BMI) according to the German reference data set [14] were visualized using scatterplots with a local polynomial regression line. We then fit univariate linear mixed models (LMM) regressing one by one each metabolite on each hormone as well as multiple models including fasting insulin, age, sex, and BMI [kg/m^2^] as potential confounders. Batch number was included as random intercept. We checked diagnostic plots to see if the assumptions of the models were met. We log-transformed the concentrations of the metabolites, leptin, and of adiponectin. To allow comparison of regression estimates we standardized our data ([observed value–mean] / SD). Variance inflation factors (VIF) were computed. We confirmed that multicollinearity was not a serious problem in the covariate-adjusted models. The results on these models are represented in the so-called Manhattan Plot: for each predictor we extracted the standardized regression coefficients of the models and plotted them on the y-axis. The color of the point is used to indicate the significance of the association between the predictor and the metabolite outcome. We applied the Bonferroni correction to counteract the problem of multiple comparisons (Bonferroni corrected significance level: α/200 = 0.00025) and we report the Bonferroni corrected P-values (*P*_BF_) in the following. Random forests analysis was performed as sensitivity analysis since metabolites were entered individually in the underlying linear models to avoid multicollinearity. All statistical analyses were performed using the statistical software R.

## Results

**Fig 1:**
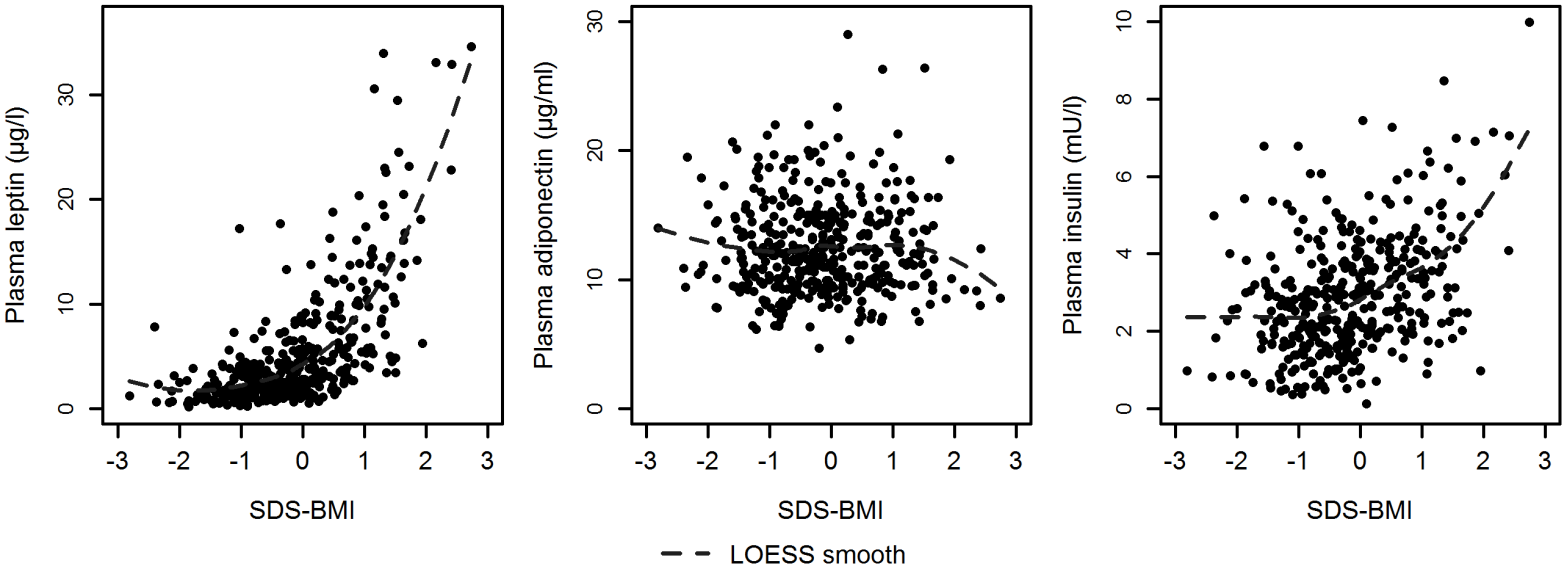
The scatter plot of SDS-BMI versus leptin, adiponectin, and insulin plasma concentrations provided along with a LOESS (locally fitted regression) line.

Adipokine and metabolites measurements were available for 400 pre-pubertal children with a mean age of 8.2 years. Characteristics of these children are presented in Table 1. Only 34 children (8%) were overweight and none was obese; 66% of the children had a SDS-BMI between -1.1 and +0.8. The HOMA index was below 2.0 for all but two children who had values of 2.7 and 2.9. The relation of the adipokines with the SDS-BMI is shown in Fig 1. While leptin and insulin showed a strong relationship with the SDS-BMI (leptin: Spearman’s ρ, 0.68; Bonferroni corrected P-value [*P*_BF_] < 0.001; insulin: Spearman’s ρ, 0.36; *P*_BF_ < 0.001), adiponectin was not associated to the SDS-BMI. These associations were similar in girls and boys.

**Table 1:** Baseline characteristics of the study population. Data are given as mean ± SD unless otherwise indicated.

Characteristics	Number of children N = 400
Female, N (%)	207 (52%)
Age (yrs)	8.2 ± 0.16
Weight (kg)	28.0 ± 4.90
Height (cm)	131.4 ± 5.34
SDS-BMI^†^	-0.2 ± 0.96
BMI Categorization, N (%)^‡^	
Underweight	44 (11%)
Normal weight	322 (80%)
Overweight	34 (8%)
Plasma leptin (μg/l)*	5.2 ± 5.76
Plasma adiponectin (μg/ml) *	12.5 ± 3.68
Plasma insulin (mU/l) *	2.9 ± 1.52

^†^ Calculated according to the German reference data set [14]

^‡^ Underweight: <10^th^ percentile; normal weight: 10^th^ - 90^th^ percentile; overweight: >90^th^ percentile

* Blood withdrawal was after an overnight fast of 10 hours.

**Fig 2:**
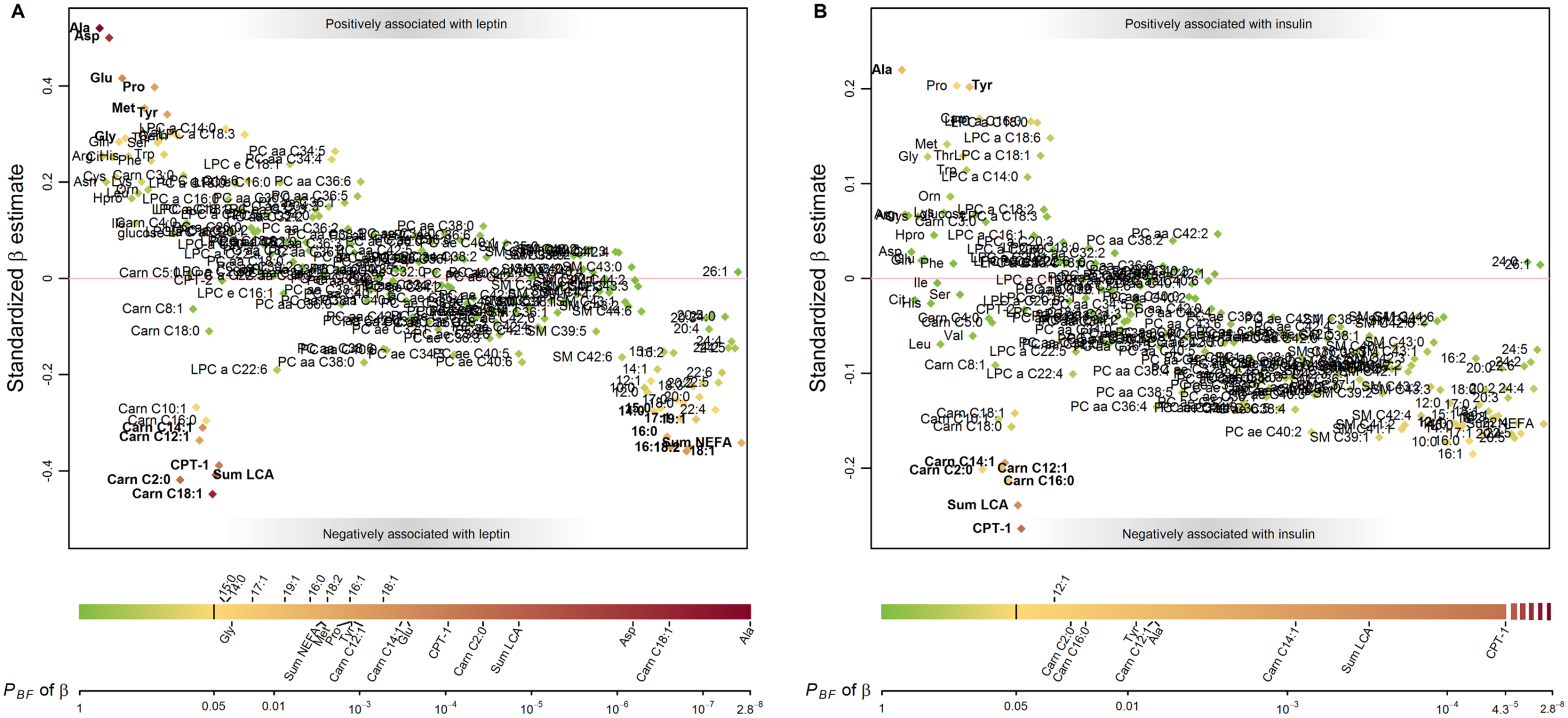
Manhattan plots for the associations of plasma fasting leptin (A) and fasting insulin (B) with plasma metabolites. Results are based on one multiple linear mixed models model for each metabolite: we regressed each metabolite on leptin, adiponectin, insulin, age, sex, and BMI and included a random intercept for batch number. Standardized β estimates (y-axis) of 200 metabolites and metabolite ratios are presented, grouped according to their chemical properties (x-axis). Estimates quantify the number of standard deviation units metabolite concentration changes with an increase in one standard deviation in log-transformed leptin and untransformed insulin concentrations. In bold are those metabolites which were significantly related to leptin / insulin. The coloring of the points indicates the Bonferroni corrected P-value [*P*_BF_] of the respective β estimate as indicated in the color bar below the plot; the black vertical line in the color bar indicates the Bonferroni corrected significance level. CPT-1 reflects the acylcarnitine ratio (C16+C18)/C0; CPT-2 reflects the acylcarnitine ratio C2/(C16+C18). Abbreviations: Carn, acylcarnitine; LCA, long-chain acylcarnitine; LPC, lysophosphatidylcholine; PCaa, diacyl-phosphatidylcholine; PCae, acyl-alkyl-phosphatidylcholine; SM, sphingomyeline; NEFA, non-esterified acid.

The results of the multiple linear mixed models (LMM) are listed in S1 Table. Adiponectin was not statistically significantly associated with any of the metabolites (S1 Fig). Fig 2 summarizes the results of the LMM with respect to leptin and insulin concentrations. The comparison of the standardized β coefficients shows that the associations between leptin and the metabolites were, in general, stronger than those for adiponectin and insulin. For instance, although alanine was among the metabolites with the strongest associations to the hormones, the standardized β was 0.52 for leptin, but only 0.22 for insulin (*P*_BF_, 8.0×10^−9^ and 0.005, respectively). The closer association of leptin to the metabolome was confirmed by random forest analysis: in contrast to fasting insulin whose variance was explained up to 16% by the metabolites, fasting leptin values were explained up to 31% (S2 Table).

Other metabolites that were associated to both leptin and insulin were tyrosine, the acylcarnitines C2, C12:1, and C14:1, the sum of the long-chain acylcarnitines and the acylcarnitine marker for CPT-1 activity defined as (C16:0+C18:0) / free carnitine, as well as the NEFA C16:1. The directions of the associations were the same for leptin and insulin: while the amino acids alanine and tyrosine showed a positive relationship to both hormones, the acylcarnitines as well as the acylcarnitine ratio and NEFA were decreased in a state of high fasting leptin and insulin concentrations. Aspartate, which was similarly strongly associated to leptin as alanine, was linked to leptin only as were the amino acids glutamate, proline, methionine, and glycine (in order of effect size). They all were positively associated to leptin. There were further statistically significant associations between metabolites and leptin or insulin concerning acylcarnitines and NEFA that follow the same pattern as the shared metabolites: leptin and insulin were inversely related to the medium- and long-chain acylcarnitines and the NEFA. We also checked for interaction effects between leptin*insulin, leptin*BMI, insulin*BMI, as well as for sex interactions leptin*sex or insulin*sex, but no interaction effect, except for leptin*insulin that negatively interacted on glucose, was significant for any metabolite at the adjusted level (S3 Table).

## Discussion

In this study, we characterized the snapshot of the intercellular and interorgan communication related to the adipocytes, adiponectin and leptin, and insulin on a molecular level. We were able to demonstrate that fasting leptin and, somehow less strong, fasting insulin levels are reflected in the metabolome. Our work contributes to the understanding of activated pathways regulating the energy homeostasis in fasting, 8 year old, pre-pubertal, and non-obese children.

The healthy children studied here are presumably metabolically similar with a limited range in BMI values. Interestingly, we nevertheless found evidence for strong correlations of the hormones leptin and insulin with selected metabolites. Adiponectin, by contrast, was unrelated to BMI and the metabolite profile.

### Associations of leptin and insulin concentrations with amino acids

Leptin plasma concentrations were positively and significantly associated with several amino acids, whereby the strongest association was observed between leptin and alanine. Alanine plays an important role in the glucose-alanine cycle transferring energy and nitrogen between muscle and liver. In the fasted state, gluconeogenesis contributes to ensure stable blood glucose levels: Glucose is formed in the liver then transported to extrahepatic tissues where it is used for glycolysis to retrieve energy. The products of this pathway, mainly lactate, alanine, or glutamine, are subsequently released and transported back to the liver for gluconeogenesis [15, 16]. Additionally, protein is catabolized for energy provision leading to the release of amino acids, primarily alanine and glutamine [17]. Considering the gluconeogenic effect of leptin [18], it is more likely that the positive association, we found between alanine and leptin concentrations, is due to this effect rather than to an increased protein breakdown in children with high leptin levels. The fact that glutamine levels, which is also a main gluconeogenic precursor in the liver, were positively but not significantly associated to plasma leptin might be due to the fact that glutamine can be formed from glutamate and ammonium by glutamine synthetase.

A look towards the other amino acids related to leptin in our study revealed that asparagine concentrations were about as strongly associated to leptin level as were alanine concentrations. Thus, our findings might also be driven by higher aminotransferase activity induced by high fasting leptin levels. Increased alanine and aspartate concentrations enhance the activation effect of pyridoxal phosphate on alanine aminotransferase (ALT) and aspartate transaminase (AST) [19, 20]. In fact, several studies found a positive relationship between the transaminases ALT and AST and leptin [21, 22]. These enzymes are furthermore commonly used as serum biomarkers for non-alcoholic fatty liver disease, a disease which already can occur in healthy children: A recent meta-analysis reported a prevalence of 7.6% in general population studies and 2.3% if furthermore restricted to normal weight children [23]. Thus, also in our healthy cohort of 8-years old children, the high association of alanine and asparagine with fasting leptin might partially be due to increased liver fat accumulation in these children.

The associations of fasting insulin with amino acids we have seen in our study fit both the gluconeogenesis and transaminase related explanation approaches. We found that insulin levels were likewise positively and significantly associated with alanine. During fasting state, the only significant NEFA liberation site is adipose tissue where NEFA arise from hydrolysis of triacylglycerol within the adipocyte [24]. Insulin exhibits antilipolytic effects. Thus, if fasting insulin levels are high, less NEFA for energy are available and the body might shift towards gluconeogenesis and use glycogen, lactate, or amino acids for energy production instead. Other studies have linked high fasting insulin levels with high transaminase concentrations. The same authors found that (hepatic) insulin resistance and type 2 diabetes were related to increased plasma ALT and AST concentration [25]. Taken together, relatively elevated fasting insulin levels might point towards a higher gluconeogenic activity, or also towards early stages of insulin resistance or fatty liver.

On this background and given our similar results on insulin and leptin, another study investigating if leptin levels reflect differences in insulin sensitivity in non-diabetic adults with a BMI ranging from 20 to 78 kg/m^2^ becomes of interest [26]. They found that insulin sensitivity explains about 40% of the variance in fasting leptin levels and that the relationship between clamp-derived insulin sensitivity and fasting leptin levels is actually more robust than that between the former and fasting insulin levels. As the ß-estimates in our study and the results from the random forest analysis also point towards a stronger association of leptin to the metabolome compared to insulin to the metabolome, this finding of ours might be explained by early stages of insulin resistance or fatty liver in some children. However, this hypothesis remains speculative as the children studied are healthy and non-obese, while the cited study was performed on adults including morbidly obese adults.

### Associations of leptin concentrations with NEFA and acylcarnitines

We found that plasma NEFA as well as long-chain acylcarnitines and acetylcarnitine levels were negatively associated with plasma leptin concentrations highlighting the involvement of leptin in the regulation of fatty acid oxidation. In fact, leptin has been shown to stimulate fatty acid oxidation in skeletal muscle by disinhibiting CPT-1 [27]. CPT-1 is the enzyme responsible for the formation of acylcarnitines from free carnitine and acyl-CoA, the product of FA activation [28]. On the other hand, leptin was shown to exhibit lipolytic effects on adipocytes incubated with leptin in vitro [29, 30] and on adipocytes in mice injected with leptin [31]. However, the lipolytic effect is small compared to adrenergic factors [32] which together with glucagon and insulin on the antilipolytic side are major regulators of lipolysis. Furthermore, the results of those studies are only partially transferable to our findings, as we investigated associations of NEFA and leptin concentrations in plasma. There are only few studies available that have investigated associations of plasma concentrations and these studies found contradictory results. One study in healthy adults found a weak and positive relationship between plasma leptin and plasma NEFA which, however, was only statistically significant in univariate analysis [33]. Other studies exploring the effect of leptin injections in animals, found that leptin reduced plasma NEFA levels in female rats [34] but increased in pigs [35]. Buettner et al. [36] investigated the sympathetic effect of leptin and found that leptin infused into the mediobasal hypothalamus of rats inhibited white adipose tissue lipogenesis. In summary, more research is needed on the relationship between leptin and NEFA. Our results depicting an inverse relationship between leptin and NEFA levels might thus be explained by a higher usage of fatty acids induced by higher CPT-1 activity stimulated by leptin. The lipolytic effect of leptin per se was not visible, possibly because it was too small or because the NEFA are directly taken up by tissue for oxidation.

Our results on acylcarnitines underpin our explanatory approach that the decreased NEFA are the result of enhanced CPT-1 activity induced by high leptin levels. We found that only long-chain acylcarnitines of length C12-C18:1 were negatively associated with leptin concentrations. In contrast to acylcarnitines of shorter length, the long-chain acylcarnitines mainly depend on the carnitine shuttle to enter the mitochondria for oxidation [12, 13]. Therefore, it is likely that short- and medium-chain acylcarnitines are less affected by CPT-1 activity.

The direction of the associations between leptin and acylcarnitine concentrations—which were inversely related to each other—needs further consideration: Acylcarnitines (being essential in fatty acid oxidation) had a lower concentration when leptin (known to enhance CPT-1 activity and formation of acylcarnitines) were high. We also calculated the acylcarnitine ratio (C16+C18)/C0 that is used to characterize the initiation rate of fatty acid oxidation and CPT-1A deficiency diagnosis in newborn screening [37]. Strikingly, this ratio also showed an inverse relationship with leptin. Acylcarnitines are formed at the outer mitochondrial membrane for transport of the FA across the mitochondrial matrix. Up to date, the physiological role of acylcarnitine efflux to the plasma compartment is unknown [38]. We can think of two possible explanations for our finding on an inverse relationship between leptin and the acylcarnitines. Lower fasting plasma acylcarnitines might indicate a higher fatty acid oxidation because NEFA (as acylcarnitines) are used for energy provision and the flux of acylcarnitines in the mitochondria is enhanced, resulting in a lower concentration in the plasma. Or, the stimulating effect of leptin on fatty acid oxidation is not reflected in plasma acylcarnitine concentrations but is only seen in cytosolic or mitochondrial acylcarnitine levels.

### Associations of insulin concentrations with NEFA and acylcarnitines

Insulin showed a similar association to acylcarnitines and NEFA as leptin. Plasma insulin concentrations were, with very few exceptions, negatively but not significantly associated with plasma NEFA and acylcarnitines in our study. The finding on the associations with NEFA is in line with the literature as insulin is known to inhibit lipolysis through suppression of HSL activation [39]. However, other studies investigating associations between plasma insulin and NEFA did not find associations between fasting insulin concentrations and NEFA concentrations, neither in normal weight or obese children [40, 41] nor consistently in healthy adults [40, 42]. Especially striking is the discrepancy to the results of Salgin et al. [40] who studied 8-years old children of the ALSPAC cohort study, with similar characteristics as in our study population. Although they found an inverse relationship between plasma NEFA and insulin secretion, they failed to prove an association between NEFA and insulin levels. In adult men, they reported a non-linear relationship between NEFA and insulin levels. In our study, there was no trend towards non-linear relationships. Thus, the negative association between fasting NEFA and fasting insulin levels is probably a result of the antilipolytic effect of insulin.

The associations we found for insulin with acylcarnitines were negative and especially strong for the long-chain acylcarnitines. We suggest these findings should be discussed in the same way as the relations of acylcarnitines with leptin: Higher fasting insulin levels seem to be related to decreased lipolysis and decreased fatty acid oxidation while the inverse relationship should make us reflect on the meaning of acylcarnitines in plasma.

Two important findings of our study are that insulin and leptin showed similar association patterns to the metabolome and that fasting leptin concentrations were more closely related to the metabolome than were insulin concentrations. Regarding the shared associations, it is important to note that our results refer to associations found in the fasting state in relatively healthy children. Based on our findings we can conclude that a higher gluconeogenesis and a higher fatty acid oxidation may go on in children with higher fasting insulin and leptin levels. Notably, the cross-sectional study design does not allow conclusions on causality. It remains unclear if the hormones shape the metabolite profile, or vice versa, or if both are a result from a third factor not assessed in this study. The second point, the stronger association of leptin to the metabolome, was evidenced by both the ß-estimates and the results from the random forest. Also with respect to the association to SDS-BMI, leptin showed stronger correlations than insulin. We briefly mentioned above that this might be related to early stages of insulin resistance in some children. However, particular care is needed as our children are metabolically healthy.

### Interactions between leptin and insulin concentrations

There is substantial evidence that leptin decreases insulin secretion [32]. Several other studies have proved an interaction between insulin and leptin when administered simultaneously [30, 43]. The examination of the interaction effects in our study revealed that leptin and insulin interacted negatively on fasting glucose. All other interaction effects between the hormones themselves or between the hormones and sex or BMI were not significant. Hence, although in vitro or in vivo studies have demonstrated interacting effects of the hormones, we did not find interaction effects in humans during fasting when energy homeostasis was reached.

### Adiponectin

In our large cohort in pre-pubertal healthy children, plasma concentrations of adiponectin did not depend on the child’s BMI which is in contrast to the literature [44–49]. We furthermore found no significant association between adiponectin and any metabolite. There are several explanations for our finding. Firstly, we measured total adiponectin. HMW-adiponectin might yield other results. Secondly, our results are based on a relatively healthy population: Only 8% of the children were overweight and none were obese which is in contrast to the above cited studies. Interestingly, although our cohort mainly consists of metabolically healthy children, we observed associations for leptin which we furthermore found to be exponentially related to BMI. The picture appears to be different for adiponectin. The narrow range of BMI and the narrow spread of adiponectin levels (mean± SD, 12.5±3.68) may explain why we did not find any association of adiponectin to BMI, a metabolite, or leptin. Adiponectin might only modulate the metabolic responses in children with already manifest obesity. Further research with special focus on overweight and obese children and HMW-adiponectin measurements is needed in order to elucidate the relationship of adiponectin to the metabolome in children.

### Strengths and limitations

A strength certainly is that we provide a comprehensive overview of the interplay of the hormones leptin, adiponectin and insulin with human metabolism. We measured a wide range of metabolites, including acylcarnitines and free fatty acids. However, we should be aware that we measured plasma concentrations which may not necessarily reflect intracellular processes. Metabolomics gives an instantaneous snapshot of the metabolism and cannot be translated into a dynamic map of metabolite traffic on biochemical routes. Another drawback might be that we possibly are missing some important measurements such as transaminases or α-ketoacids. Thus, we suggest that further studies should focus on metabolites involved in gluconeogenesis, lipolysis, and fatty acid oxidation and directly measure the activity of pathways or the concentration of enzymes involved in the respective pathway. With respect to our study population, the loss to follow-up may have distorted our birth cohort and selected the highly motivated participants. Nevertheless, we had available adipokine and metabolomics measurements for 38% of the initial study participants. The last aspect we would like to highlight is the healthy nature of our cohort: while, on the one hand, this is a clear advantage as we can rule out that our children were insulin resistant, it constitutes a limitation as some pathways may become affected in obese children only. Thus, our speculations on early stages of metabolic derangements should be tested in studies with different population characteristics.

## Conclusion

Based on data from healthy, 8-year old children of the Ulm Birth Cohort Study, we describe associations of hormones leptin and insulin to pathways activated in the fasting state and explore the interrelations of those hormones. We show that leptin levels are strongly reflected in the metabolic profile. Associations to insulin are similar but less strong. Fasting concentrations of both hormones seem related to energy providing pathways in the fasting state, namely higher gluconeogenesis and fatty acid oxidation. Hormone concentrations had no co-regulating effect on the metabolism in the fasting state, except for glucose metabolism. Both, fasting leptin and insulin concentrations, seem to be independently related to catabolic metabolic processes.

## Supporting information

S1 Fig Manhattan plot for the associations of plasma adiponectin with plasma metabolites. Results are based on one multiple linear mixed models model for each metabolite: we regressed each metabolite on leptin, adiponectin, insulin, age, sex, and BMI and included a random intercept for batch number. Standardized β coefficients (y-axis) of 200 metabolites and metabolite ratios are presented, grouped according to their chemical properties (x-axis). The coloring of the points indicates the Bonferroni corrected *P*_BF_ = log_10_(*P*) of the respective β coefficient; the black vertical line in the color bar indicates the Bonferroni corrected significance level. CPT-1 reflects the acylcarnitine ratio (C16+C18)/C0; CPT-2 reflects the acylcarnitine ratio C2/(C16+C18). Abbreviations: Carn, acylcarnitine; LCA, long-chain acylcarnitine; LPC, lysophosphatidylcholine; PCaa, diacyl-phosphatidylcholine; PCae, acyl-alkyl-phosphatidylcholine; SM, sphingomyeline; NEFA, non-esterified acid.
(TIFF)

S1 Table Results of the linear mixed models (LMM) regressing each metabolite on fasting leptin, fasting adiponectin, fasting insulin, age, sex, and BMI with a random intercept for batch number. The standardized regression coefficient β and the Bonferroni corrected P value [*P*_BF_] are given for each independent variable. Additionally, we indicate the global mean (SD) in μmol/L of the metabolites in the whole sample.
(DOCX)

S2 Table Variable importance as assessed by the random forests on leptin, adiponectin, and insulin. Variable selection was done by successively eliminating 20% of the least important variables. The mean squared error (MSE) was calculated using 10-fold cross validation and indicates the average of the squares of the prediction errors using the respective number of variables.
(DOCX)

S3 Table Results of the linear mixed models (LMM) regressing each metabolite on fasting leptin, fasting adiponectin, fasting insulin, age, sex, BMI, and the respective interaction terms (added one by one), with a random intercept for batch number. The standardized regression coefficient β and the Bonferroni corrected P value [*P*_BF_] are given for each interaction effect. CPT-1 reflects the acylcarnitine ratio (C16+C18)/C0; CPT-2 reflects the acylcarnitine ratio (C2/(C16+C18)).
(DOCX)

## Abbreviations

AA: amino acids
ALT: alanine aminotransferase
AST: aspartate transaminase
CPT-1: carnitine-palmitoyltransferase-1
LMM: linear mixed effects model
LPCa: acyl- lysophosphatidylcholines
LPCe: alkyl-lysophosphatidylcholines
NEFA: non-esterified fatty acids
PCaa: diacyl-phosphatidylcholines
PCae: acyl-alkyl-phosphatidylcholines
SM: sphingomyelines
UBCS: Ulm Birth Cohort Study
