# Supplementary figures and images for: Metabolomics reveals an entanglement of fasting leptin concentrations with fatty acid oxidation and gluconeogenesis in healthy children
Source: PLoS One. 2017 Aug 17;12(8):e0183185. doi: 10.1371/journal.pone.0183185 (PMC5560563; doi:10.1371/journal.pone.0183185)

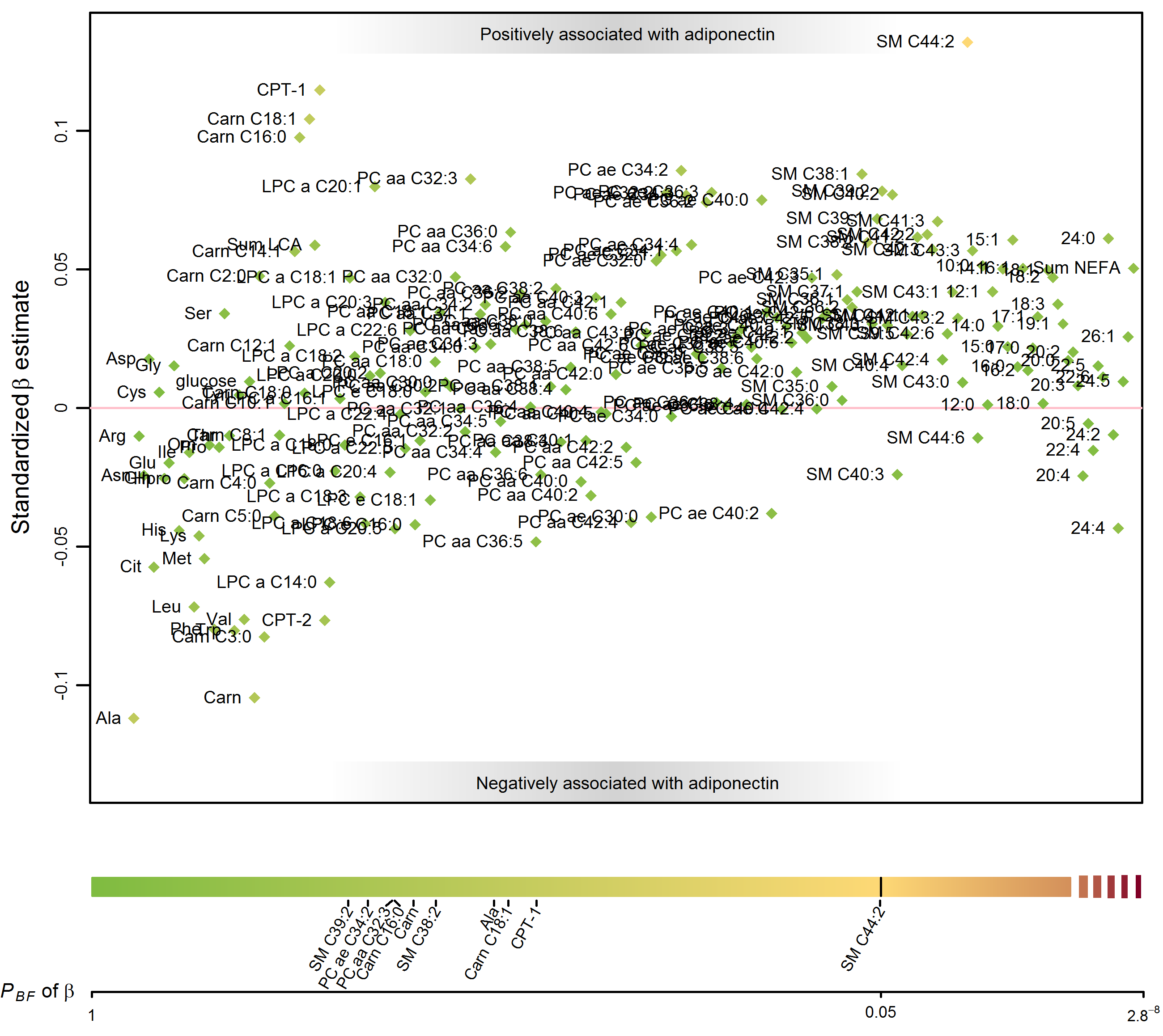

Supplement: S1 Fig — Results are based on one multiple linear mixed models model for each metabolite: we regressed each metabolite on leptin, adiponectin, insulin, age, sex, and BMI and included a random intercept for batch number. Standardized β coefficients (y-axis) of 200 metabolites and metabolite ratios are presented, grouped according to their chemical properties (x-axis). The coloring of the points indicates the Bonferroni corrected PBF = log10(P) of the respective β coefficient; the black vertical line in the color bar indicates the Bonferroni corrected significance level. CPT-1 reflects the acylcarnitine ratio (C16+C18)/C0; CPT-2 reflects the acylcarnitine ratio C2/(C16+C18). Abbreviations: Carn, acylcarnitine; LCA, long-chain acylcarnitine; LPC, lysophosphatidylcholine; PCaa, diacyl-phosphatidylcholine; PCae, acyl-alkyl-phosphatidylcholine; SM, sphingomyeline; NEFA, non-esterified acid. (TIFF) [file pone.0183185.s001.tiff]
