# Supplementary material for: Metabolomics reveals an entanglement of fasting leptin concentrations with fatty acid oxidation and gluconeogenesis in healthy children
Source: PLoS One. 2017 Aug 17;12(8):e0183185. doi: 10.1371/journal.pone.0183185 (PMC5560563; doi:10.1371/journal.pone.0183185)
Supplement: S1 Table — The standardized regression coefficient β and the Bonferroni corrected P value [PBF] are given for each independent variable. Additionally, we indicate the global mean (SD) in μmol/L of the metabolites in the whole sample. (DOCX) [file pone.0183185.s002.docx]

**S1 Table.** Results of the linear mixed models (LMM) regressing each metabolite on fasting leptin, fasting adiponectin, fasting insulin, age, sex, and BMI with a random intercept for batch number. The standardized regression coefficient β and the Bonferroni corrected P value [*P*_BF_] are given for each independent variable. Additionally, we indicate the global mean (SD) in µmol/L of the metabolites in the whole sample.

| **Metabolite (Outcome)** | **Mean (SD)** | **Leptin** | |  | **Adiponectin** | |  | **Insulin** | |  | **Age** | |  | **Sex** | |  | **BMI** | |
| --- | --- | --- | --- | --- | --- | --- | --- | --- | --- | --- | --- | --- | --- | --- | --- | --- | --- | --- |
|  |  | **β** | ***P*_BF_** |  | **β** | ***P*_BF_** |  | **β** | ***P*_BF_** |  | **β** | ***P*_BF_** |  | **β** | ***P*_BF_** |  | **β** | ***P*_BF_** |
| Ala | 347.1 (81.4) | 0.52 | 8.0×10^-9^ |  | -0.11 | 1 |  | 0.22 | 0.005 |  | 0.015 | 1 |  | -0.14 | 1 |  | -0.32 | 0.003 |
| Arg | 83.0 (27.6) | 0.25 | 0.75 |  | -0.01 | 1 |  | 0.067 | 1 |  | 0.015 | 1 |  | -0.2 | 1 |  | -0.25 | 0.42 |
| Asn | 49.7 (9.61) | 0.2 | 1 |  | -0.024 | 1 |  | 0.066 | 1 |  | 0.023 | 1 |  | -0.065 | 1 |  | -0.19 | 1 |
| Asp | 17.0 (4.99) | 0.5 | 2.4×10^-7^ |  | 0.018 | 1 |  | 0.028 | 1 |  | 0.018 | 1 |  | -0.085 | 1 |  | -0.26 | 0.17 |
| Cit | 33.8 (8.36) | 0.25 | 0.61 |  | -0.057 | 1 |  | -0.023 | 1 |  | -0.031 | 1 |  | -0.12 | 1 |  | -0.27 | 0.1 |
| Cys | 30.6 (7.72) | 0.21 | 0.74 |  | 0.0056 | 1 |  | 0.065 | 1 |  | -0.05 | 1 |  | -0.19 | 1 |  | -0.015 | 1 |
| Gln | 594.8 (88.3) | 0.28 | 0.14 |  | -0.025 | 1 |  | 0.019 | 1 |  | -0.045 | 1 |  | 0.13 | 1 |  | -0.27 | 0.11 |
| Glu | 84.6 (17.3) | 0.42 | 1.8×10^-4^ |  | -0.02 | 1 |  | 0.021 | 1 |  | 0.016 | 1 |  | -0.21 | 1 |  | -0.18 | 1 |
| Gly | 265.0 (50.4) | 0.29 | 0.026 |  | 0.015 | 1 |  | 0.13 | 1 |  | 0.054 | 1 |  | -0.067 | 1 |  | -0.34 | 4.3×10^-4^ |
| His | 111.7 (19.9) | 0.25 | 0.29 |  | -0.044 | 1 |  | -0.026 | 1 |  | -0.002 | 1 |  | 0.097 | 1 |  | -0.075 | 1 |
| Hpro | 23.8 (10.1) | 0.17 | 1 |  | -0.026 | 1 |  | 0.046 | 1 |  | 0.0071 | 1 |  | -0.069 | 1 |  | -0.12 | 1 |
| Ile | 64.2 (11.8) | 0.11 | 1 |  | -0.016 | 1 |  | -0.0048 | 1 |  | -0.02 | 1 |  | -0.014 | 1 |  | -0.029 | 1 |
| Leu | 131.2 (21.7) | 0.18 | 1 |  | -0.072 | 1 |  | -0.07 | 1 |  | -0.024 | 1 |  | -0.05 | 1 |  | 0.041 | 1 |
| Lys | 169.9 (33.5) | 0.2 | 0.65 |  | -0.046 | 1 |  | 0.069 | 1 |  | 0.0029 | 1 |  | 0.23 | 1 |  | -0.085 | 1 |
| Met | 25.7 (5.47) | 0.35 | 0.002 |  | -0.054 | 1 |  | 0.14 | 1 |  | -0.023 | 1 |  | -0.035 | 1 |  | -0.28 | 0.038 |
| Orn | 64.7 (16.2) | 0.18 | 1 |  | -0.013 | 1 |  | 0.086 | 1 |  | -0.013 | 1 |  | 0.23 | 1 |  | -0.19 | 1 |
| Phe | 62.8 (10.6) | 0.24 | 0.46 |  | -0.08 | 1 |  | 0.016 | 1 |  | -0.018 | 1 |  | -0.034 | 1 |  | -0.088 | 1 |
| Pro | 223.4 (70.6) | 0.4 | 0.001 |  | -0.014 | 1 |  | 0.2 | 0.07 |  | 0.0032 | 1 |  | -0.29 | 1 |  | -0.3 | 0.043 |
| Ser | 150.8 (35.7) | 0.28 | 0.15 |  | 0.034 | 1 |  | -0.017 | 1 |  | 0.027 | 1 |  | 0.0056 | 1 |  | -0.31 | 0.014 |
| Thr | 120.6 (27.3) | 0.29 | 0.07 |  | -0.01 | 1 |  | 0.13 | 1 |  | -0.0061 | 1 |  | -0.13 | 1 |  | -0.22 | 0.8 |
| Trp | 69.4 (13.3) | 0.26 | 0.29 |  | -0.08 | 1 |  | 0.11 | 1 |  | 0.033 | 1 |  | -0.082 | 1 |  | -0.12 | 1 |
| Tyr | 74.8 (15.4) | 0.34 | 0.001 |  | 0.0046 | 1 |  | 0.2 | 0.007 |  | -0.018 | 1 |  | -0.083 | 1 |  | -0.084 | 1 |
| Val | 249.7 (42.5) | 0.3 | 0.06 |  | -0.076 | 1 |  | -0.061 | 1 |  | -0.014 | 1 |  | -0.069 | 1 |  | 0.002 | 1 |
| Glucose | 87.7 (9.27) | 0.094 | 1 |  | 0.0096 | 1 |  | 0.068 | 1 |  | 0.09 | 1 |  | -0.19 | 1 |  | -0.065 | 1 |
| Carn | 32.8 (6.91) | 0.3 | 0.1 |  | -0.1 | 1 |  | 0.17 | 0.5 |  | 0.0019 | 1 |  | -0.21 | 1 |  | -0.11 | 1 |
| Carn C2:0 | 7.01 (2.69) | -0.42 | 2.1×10^-5^ |  | 0.048 | 1 |  | -0.2 | 0.019 |  | 0.032 | 1 |  | 0.19 | 1 |  | 0.29 | 0.018 |
| Carn C3:0 | 0.39 (0.13) | 0.21 | 1 |  | -0.083 | 1 |  | 0.06 | 1 |  | -0.0073 | 1 |  | -0.18 | 1 |  | -0.082 | 1 |
| Carn C4:0 | 0.2 (0.082) | 0.12 | 1 |  | -0.027 | 1 |  | -0.042 | 1 |  | -0.058 | 1 |  | -0.2 | 1 |  | -0.065 | 1 |
| Carn C5:0 | 0.11 (0.05) | 0.011 | 1 |  | -0.039 | 1 |  | -0.046 | 1 |  | -0.056 | 1 |  | -0.14 | 1 |  | 0.049 | 1 |
| Carn C8:1 | 0.21 (0.14) | -0.063 | 1 |  | -0.0098 | 1 |  | -0.091 | 1 |  | 0.078 | 1 |  | 0.024 | 1 |  | 0.17 | 1 |
| Carn C10:1 | 0.29 (0.15) | -0.27 | 0.09 |  | 0.0017 | 1 |  | -0.15 | 0.57 |  | 0.086 | 1 |  | 0.35 | 0.055 |  | 0.19 | 1 |
| Carn C12:1 | 0.26 (0.15) | -0.34 | 0.001 |  | 0.022 | 1 |  | -0.2 | 0.006 |  | 0.041 | 1 |  | 0.4 | 0.004 |  | 0.23 | 0.12 |
| Carn C14:1 | 0.3 (0.11) | -0.31 | 2.2×10^-4^ |  | 0.056 | 1 |  | -0.19 | 0.001 |  | 0.039 | 1 |  | 0.37 | 0.001 |  | 0.28 | 4.5×10^-4^ |
| Carn C16:0 | 0.1 (0.043) | -0.3 | 0.07 |  | 0.098 | 1 |  | -0.21 | 0.015 |  | -0.024 | 1 |  | 0.16 | 1 |  | 0.21 | 1 |
| Carn C18:0 | 0.045 (0.02) | -0.11 | 1 |  | 0.0053 | 1 |  | -0.16 | 1 |  | -0.00082 | 1 |  | 0.025 | 1 |  | 0.0092 | 1 |
| Carn C18:1 | 0.14 (0.05) | -0.45 | 8.9×10^-8^ |  | 0.1 | 1 |  | -0.14 | 0.51 |  | 0.024 | 1 |  | 0.38 | 0.006 |  | 0.33 | 1.5×10^-4^ |
| Sum LCA | 0.86 (0.32) | -0.41 | 7.4×10^-6^ |  | 0.059 | 1 |  | -0.24 | 2.0×10^-4^ |  | 0.031 | 1 |  | 0.41 | 0.003 |  | 0.32 | 0.001 |
| CPT-1 | 0.0047 (0.0023) | -0.39 | 5.7×10^-5^ |  | 0.11 | 1 |  | -0.26 | 2.5×10^-5^ |  | -0.018 | 1 |  | 0.21 | 1 |  | 0.21 | 0.65 |
| CPT-2 | 29.7 (9.26) | -0.0045 | 1 |  | -0.077 | 1 |  | -0.033 | 1 |  | 0.041 | 1 |  | -0.14 | 1 |  | -0.01 | 1 |
| LPC a C14:0 | 1.23 (0.48) | 0.31 | 0.047 |  | -0.063 | 1 |  | 0.11 | 1 |  | 0.0087 | 1 |  | -0.16 | 1 |  | -0.022 | 1 |
| LPC a C16:0 | 56.6 (12.4) | 0.16 | 1 |  | -0.023 | 1 |  | 0.17 | 0.36 |  | 0.089 | 1 |  | -0.037 | 1 |  | -0.087 | 1 |
| LPC a C16:1 | 1.86 (0.51) | 0.096 | 1 |  | 0.0034 | 1 |  | 0.045 | 1 |  | 0.081 | 1 |  | 0.069 | 1 |  | 0.13 | 1 |
| LPC a C18:0 | 20.0 (4.72) | 0.2 | 1 |  | -0.013 | 1 |  | 0.16 | 0.49 |  | 0.071 | 1 |  | -0.073 | 1 |  | -0.13 | 1 |
| LPC a C18:1 | 14.7 (3.57) | 0.14 | 1 |  | 0.047 | 1 |  | 0.13 | 1 |  | 0.079 | 1 |  | -0.026 | 1 |  | -0.16 | 1 |
| LPC a C18:2 | 24.5 (7.4) | 0.14 | 1 |  | 0.019 | 1 |  | 0.072 | 1 |  | 0.072 | 1 |  | 0.0058 | 1 |  | -0.13 | 1 |
| LPC a C18:3 | 0.51 (0.19) | 0.3 | 0.1 |  | -0.032 | 1 |  | 0.065 | 1 |  | 0.064 | 1 |  | -0.19 | 1 |  | -0.13 | 1 |
| LPC a C18:6 | 0.23 (0.072) | 0.2 | 1 |  | -0.042 | 1 |  | 0.15 | 1 |  | 0.15 | 1 |  | -0.046 | 1 |  | -0.15 | 1 |
| LPC a C20:0 | 0.2 (0.071) | 0.11 | 1 |  | 0.012 | 1 |  | 0.03 | 1 |  | 0.093 | 1 |  | -0.19 | 1 |  | -0.15 | 1 |
| LPC a C20:1 | 0.27 (0.08) | 0.096 | 1 |  | 0.08 | 1 |  | 0.022 | 1 |  | -0.04 | 1 |  | -0.21 | 1 |  | -0.098 | 1 |
| LPC a C20:2 | 0.26 (0.086) | 0.099 | 1 |  | 0.013 | 1 |  | 0.017 | 1 |  | 0.015 | 1 |  | -0.078 | 1 |  | -0.035 | 1 |
| LPC a C20:3 | 1.77 (0.45) | 0.013 | 1 |  | 0.038 | 1 |  | 0.038 | 1 |  | 0.062 | 1 |  | -0.12 | 1 |  | 0.11 | 1 |
| LPC a C20:4 | 4.42 (1.06) | 0.07 | 1 |  | -0.023 | 1 |  | 0.016 | 1 |  | 0.15 | 0.96 |  | -0.13 | 1 |  | 0.038 | 1 |
| LPC a C20:5 | 0.41 (0.16) | 0.13 | 1 |  | -0.044 | 1 |  | -0.024 | 1 |  | 0.06 | 1 |  | -0.1 | 1 |  | 0.053 | 1 |
| LPC a C22:4 | 0.36 (0.12) | 0.05 | 1 |  | -0.0023 | 1 |  | -0.1 | 1 |  | 0.04 | 1 |  | -0.17 | 1 |  | 0.082 | 1 |
| LPC a C22:5 | 0.83 (0.22) | 0.006 | 1 |  | -0.015 | 1 |  | -0.077 | 1 |  | 0.069 | 1 |  | -0.069 | 1 |  | 0.05 | 1 |
| LPC a C22:6 | 1.28 (0.36) | -0.19 | 1 |  | 0.028 | 1 |  | 0.018 | 1 |  | 0.06 | 1 |  | -0.041 | 1 |  | 0.12 | 1 |
| LPC e C16:0 | 0.7 (0.2) | 0.2 | 1 |  | -0.042 | 1 |  | -0.002 | 1 |  | 0.073 | 1 |  | -0.0026 | 1 |  | -0.075 | 1 |
| LPC e C16:1 | 0.058 (0.024) | -0.031 | 1 |  | -0.012 | 1 |  | -0.021 | 1 |  | -0.015 | 1 |  | 0.11 | 1 |  | 0.17 | 1 |
| LPC e C18:0 | 1.03 (0.27) | 0.077 | 1 |  | 0.0057 | 1 |  | 0.032 | 1 |  | 0.11 | 1 |  | -0.11 | 1 |  | -0.14 | 1 |
| LPC e C18:1 | 0.26 (0.074) | 0.24 | 1 |  | -0.033 | 1 |  | -0.036 | 1 |  | 0.071 | 1 |  | -0.17 | 1 |  | -0.11 | 1 |
| PC aa C18:0 | 0.15 (0.044) | 0.035 | 1 |  | 0.017 | 1 |  | 0.016 | 1 |  | 0.029 | 1 |  | -0.084 | 1 |  | -0.072 | 1 |
| PC aa C18:1 | 0.1 (0.038) | 0.075 | 1 |  | 0.035 | 1 |  | -0.042 | 1 |  | -0.053 | 1 |  | -0.15 | 1 |  | -0.17 | 1 |
| PC aa C30:0 | 3.70 (1.57) | 0.17 | 1 |  | 0.0091 | 1 |  | -0.012 | 1 |  | -0.028 | 1 |  | -0.13 | 1 |  | -0.0069 | 1 |
| PC aa C30:2 | 1.43 (0.47) | 0.023 | 1 |  | 0.0078 | 1 |  | -0.068 | 1 |  | 0.028 | 1 |  | 0.11 | 1 |  | 0.03 | 1 |
| PC aa C32:0 | 19.1 (5.76) | 0.075 | 1 |  | 0.047 | 1 |  | -0.07 | 1 |  | 0.03 | 1 |  | -0.03 | 1 |  | -0.053 | 1 |
| PC aa C32:1 | 17.6 (6.48) | 0.058 | 1 |  | -0.00041 | 1 |  | -0.012 | 1 |  | 0.027 | 1 |  | 0.047 | 1 |  | 0.11 | 1 |
| PC aa C32:2 | 3.92 (1.65) | 0.13 | 1 |  | -0.0085 | 1 |  | 0.027 | 1 |  | -0.0022 | 1 |  | -0.0087 | 1 |  | 0.091 | 1 |
| PC aa C32:3 | 0.51 (0.17) | 0.14 | 1 |  | 0.083 | 1 |  | -0.04 | 1 |  | 0.017 | 1 |  | 0.19 | 1 |  | 0.067 | 1 |
| PC aa C34:0 | 5.16 (2.18) | 0.13 | 1 |  | 0.022 | 1 |  | -0.054 | 1 |  | -0.015 | 1 |  | -0.15 | 1 |  | -0.12 | 1 |
| PC aa C34:1 | 224.0 (49.8) | 0.0053 | 1 |  | 0.034 | 1 |  | -0.046 | 1 |  | 0.018 | 1 |  | 0.11 | 1 |  | -0.0068 | 1 |
| PC aa C34:2 | 404.7 (91.8) | 0.011 | 1 |  | 0.037 | 1 |  | -0.044 | 1 |  | 0.042 | 1 |  | 0.14 | 1 |  | 0.01 | 1 |
| PC aa C34:3 | 14.0 (4.39) | 0.15 | 1 |  | 0.023 | 1 |  | -0.038 | 1 |  | 0.061 | 1 |  | -0.031 | 1 |  | 0.069 | 1 |
| PC aa C34:4 | 1.81 (0.72) | 0.25 | 0.27 |  | -0.016 | 1 |  | 0.00081 | 1 |  | 0.03 | 1 |  | -0.21 | 1 |  | 0.056 | 1 |
| PC aa C34:5 | 0.22 (0.1) | 0.26 | 0.27 |  | -0.0049 | 1 |  | -0.028 | 1 |  | 0.027 | 1 |  | -0.28 | 1 |  | -0.015 | 1 |
| PC aa C34:6 | 0.11 (0.048) | 0.018 | 1 |  | 0.058 | 1 |  | -0.0083 | 1 |  | -0.0029 | 1 |  | -0.048 | 1 |  | -0.0074 | 1 |
| PC aa C36:0 | 3.12 (0.98) | -0.054 | 1 |  | 0.063 | 1 |  | -0.065 | 1 |  | -0.005 | 1 |  | -0.061 | 1 |  | 0.045 | 1 |
| PC aa C36:1 | 56.9 (16) | 0.16 | 1 |  | 0.028 | 1 |  | 0.0026 | 1 |  | 0.015 | 1 |  | -0.011 | 1 |  | -0.12 | 1 |
| PC aa C36:2 | 236.8 (44.7) | 0.1 | 1 |  | 0.041 | 1 |  | -0.0021 | 1 |  | 0.036 | 1 |  | 0.11 | 1 |  | -0.034 | 1 |
| PC aa C36:3 | 117.6 (21.8) | 0.072 | 1 |  | 0.03 | 1 |  | -0.064 | 1 |  | 0.057 | 1 |  | -0.014 | 1 |  | 0.069 | 1 |
| PC aa C36:4 | 163.8 (33.6) | 0.054 | 1 |  | 0.00026 | 1 |  | -0.14 | 1 |  | 0.13 | 1 |  | -0.11 | 1 |  | 0.11 | 1 |
| PC aa C36:5 | 13.3 (5.2) | 0.17 | 1 |  | -0.048 | 1 |  | -0.072 | 1 |  | 0.057 | 1 |  | -0.18 | 1 |  | 0.058 | 1 |
| PC aa C36:6 | 0.65 (0.24) | 0.2 | 1 |  | -0.024 | 1 |  | 0.013 | 1 |  | 0.0087 | 1 |  | -0.23 | 1 |  | 0.033 | 1 |
| PC aa C38:0 | 2.44 (0.68) | -0.17 | 1 |  | 0.031 | 1 |  | -0.058 | 1 |  | 0.012 | 1 |  | 0.0052 | 1 |  | 0.14 | 1 |
| PC aa C38:1 | 1.78 (0.66) | -0.047 | 1 |  | 0.0078 | 1 |  | 0.003 | 1 |  | 0.039 | 1 |  | -0.11 | 1 |  | 0.054 | 1 |
| PC aa C38:2 | 5.71 (1.73) | -0.024 | 1 |  | 0.043 | 1 |  | 0.04 | 1 |  | -0.054 | 1 |  | -0.033 | 1 |  | 0.025 | 1 |
| PC aa C38:3 | 36.5 (8.7) | 0.091 | 1 |  | -0.012 | 1 |  | 0.0081 | 1 |  | 0.028 | 1 |  | -0.053 | 1 |  | 0.15 | 1 |
| PC aa C38:4 | 113.3 (27.2) | -0.00097 | 1 |  | 0.0067 | 1 |  | -0.098 | 1 |  | 0.13 | 1 |  | -0.021 | 1 |  | 0.16 | 1 |
| PC aa C38:5 | 48.4 (13) | 0.0056 | 1 |  | 0.015 | 1 |  | -0.12 | 1 |  | 0.09 | 1 |  | -0.058 | 1 |  | 0.12 | 1 |
| PC aa C38:6 | 56.0 (15.8) | -0.15 | 1 |  | 0.027 | 1 |  | -0.062 | 1 |  | 0.051 | 1 |  | -0.0094 | 1 |  | 0.13 | 1 |
| PC aa C40:0 | 0.46 (0.14) | -0.15 | 1 |  | -0.027 | 1 |  | -0.019 | 1 |  | -0.018 | 1 |  | -0.05 | 1 |  | 0.076 | 1 |
| PC aa C40:1 | 0.21 (0.075) | -0.033 | 1 |  | -0.012 | 1 |  | -0.0055 | 1 |  | 0.023 | 1 |  | -0.11 | 1 |  | -0.013 | 1 |
| PC aa C40:2 | 0.23 (0.084) | -0.0052 | 1 |  | -0.032 | 1 |  | -0.021 | 1 |  | -0.022 | 1 |  | -0.025 | 1 |  | 0.032 | 1 |
| PC aa C40:3 | 0.39 (0.13) | 0.019 | 1 |  | 0.04 | 1 |  | 0.0071 | 1 |  | 0.017 | 1 |  | -0.14 | 1 |  | -0.065 | 1 |
| PC aa C40:4 | 4.21 (1.28) | 0.044 | 1 |  | -0.0015 | 1 |  | -0.058 | 1 |  | 0.024 | 1 |  | -0.044 | 1 |  | 0.053 | 1 |
| PC aa C40:5 | 10.9 (2.79) | 0.017 | 1 |  | -0.0021 | 1 |  | -0.077 | 1 |  | 0.11 | 1 |  | 0.00013 | 1 |  | 0.11 | 1 |
| PC aa C40:6 | 20.6 (5.23) | -0.046 | 1 |  | 0.034 | 1 |  | -0.0027 | 1 |  | 0.035 | 1 |  | -0.047 | 1 |  | 0.13 | 1 |
| PC aa C42:0 | 0.41 (0.15) | -0.088 | 1 |  | 0.012 | 1 |  | -0.032 | 1 |  | 0.00083 | 1 |  | -0.079 | 1 |  | 0.007 | 1 |
| PC aa C42:1 | 0.21 (0.077) | -0.08 | 1 |  | 0.038 | 1 |  | 0.0047 | 1 |  | -0.054 | 1 |  | -0.079 | 1 |  | 0.013 | 1 |
| PC aa C42:2 | 0.12 (0.047) | 0.088 | 1 |  | -0.014 | 1 |  | 0.047 | 1 |  | 0.018 | 1 |  | -0.2 | 1 |  | -0.12 | 1 |
| PC aa C42:4 | 0.18 (0.071) | 0.034 | 1 |  | -0.041 | 1 |  | -0.037 | 1 |  | 0.059 | 1 |  | 0.018 | 1 |  | -0.075 | 1 |
| PC aa C42:5 | 0.33 (0.11) | 0.058 | 1 |  | -0.02 | 1 |  | -0.068 | 1 |  | -0.016 | 1 |  | 0.016 | 1 |  | -0.03 | 1 |
| PC aa C42:6 | 0.38 (0.13) | 0.041 | 1 |  | 0.023 | 1 |  | -0.086 | 1 |  | -0.007 | 1 |  | -0.21 | 1 |  | 0.047 | 1 |
| PC aa C43:6 | 0.88 (0.35) | -0.09 | 1 |  | 0.027 | 1 |  | -0.048 | 1 |  | -0.015 | 1 |  | -0.058 | 1 |  | 0.0074 | 1 |
| PC ae C30:0 | 0.37 (0.15) | 0.076 | 1 |  | -0.039 | 1 |  | -0.067 | 1 |  | -0.038 | 1 |  | -0.088 | 1 |  | -0.02 | 1 |
| PC ae C32:0 | 3.93 (0.97) | 0.013 | 1 |  | 0.053 | 1 |  | -0.096 | 1 |  | 0.0091 | 1 |  | 0.00059 | 1 |  | -0.022 | 1 |
| PC ae C32:1 | 2.74 (0.73) | -0.11 | 1 |  | 0.055 | 1 |  | -0.14 | 1 |  | 0.024 | 1 |  | 0.044 | 1 |  | 0.14 | 1 |
| PC ae C32:2 | 0.42 (0.13) | -0.014 | 1 |  | 0.078 | 1 |  | -0.13 | 1 |  | 0.061 | 1 |  | 0.19 | 1 |  | 0.11 | 1 |
| PC ae C34:0 | 2.17 (0.72) | 0.093 | 1 |  | -0.0031 | 1 |  | -0.14 | 1 |  | 0.025 | 1 |  | -0.0096 | 1 |  | -0.059 | 1 |
| PC ae C34:1 | 10.4 (2.34) | -0.016 | 1 |  | 0.057 | 1 |  | -0.084 | 1 |  | 0.026 | 1 |  | 0.053 | 1 |  | -0.031 | 1 |
| PC ae C34:2 | 11.8 (3.19) | -0.019 | 1 |  | 0.086 | 1 |  | -0.059 | 1 |  | 0.0094 | 1 |  | 0.084 | 1 |  | 0.067 | 1 |
| PC ae C34:3 | 9.58 (2.78) | -0.16 | 1 |  | 0.077 | 1 |  | -0.11 | 1 |  | 0.014 | 1 |  | 0.11 | 1 |  | 0.2 | 0.65 |
| PC ae C34:4 | 0.23 (0.092) | 0.043 | 1 |  | 0.059 | 1 |  | -0.1 | 1 |  | 0.03 | 1 |  | -0.019 | 1 |  | 0.13 | 1 |
| PC ae C36:0 | 0.92 (0.29) | 0.079 | 1 |  | 0.019 | 1 |  | -0.11 | 1 |  | -0.043 | 1 |  | 0.022 | 1 |  | -0.044 | 1 |
| PC ae C36:1 | 4.87 (1.3) | 0.039 | 1 |  | 0.018 | 1 |  | -0.12 | 1 |  | -0.021 | 1 |  | 0.079 | 1 |  | -0.092 | 1 |
| PC ae C36:2 | 11.7 (2.57) | -0.052 | 1 |  | 0.074 | 1 |  | -0.12 | 1 |  | 0.044 | 1 |  | 0.076 | 1 |  | -0.037 | 1 |
| PC ae C36:3 | 7.63 (1.82) | -0.092 | 1 |  | 0.078 | 1 |  | -0.041 | 1 |  | -0.018 | 1 |  | 0.033 | 1 |  | 0.1 | 1 |
| PC ae C36:4 | 19.5 (4.87) | -0.041 | 1 |  | 0.0019 | 1 |  | -0.09 | 1 |  | -0.00016 | 1 |  | -0.091 | 1 |  | 0.14 | 1 |
| PC ae C36:5 | 12.8 (3.32) | -0.079 | 1 |  | 0.014 | 1 |  | -0.14 | 1 |  | 0.058 | 1 |  | -0.027 | 1 |  | 0.22 | 1 |
| PC ae C36:6 | 0.45 (0.22) | 0.089 | 1 |  | 0.00067 | 1 |  | -0.057 | 1 |  | -0.018 | 1 |  | -0.15 | 1 |  | 0.039 | 1 |
| PC ae C38:0 | 1.54 (0.51) | 0.11 | 1 |  | 0.023 | 1 |  | -0.11 | 1 |  | 0.041 | 1 |  | -0.0099 | 1 |  | 0.044 | 1 |
| PC ae C38:2 | 2.29 (0.55) | 0.044 | 1 |  | 0.026 | 1 |  | -0.062 | 1 |  | -0.089 | 1 |  | 0.0082 | 1 |  | -0.052 | 1 |
| PC ae C38:3 | 4.07 (1.05) | -0.12 | 1 |  | 0.029 | 1 |  | -0.1 | 1 |  | -0.005 | 1 |  | -0.022 | 1 |  | 0.1 | 1 |
| PC ae C38:4 | 17.2 (4.14) | -0.069 | 1 |  | 0.0012 | 1 |  | -0.14 | 1 |  | 0.0038 | 1 |  | -0.075 | 1 |  | 0.034 | 1 |
| PC ae C38:5 | 20.9 (5.23) | -0.092 | 1 |  | 0.022 | 1 |  | -0.062 | 1 |  | 0.058 | 1 |  | -0.052 | 1 |  | 0.11 | 1 |
| PC ae C38:6 | 7.85 (2.39) | -0.12 | 1 |  | 0.018 | 1 |  | -0.084 | 1 |  | 0.021 | 1 |  | 0.036 | 1 |  | 0.2 | 0.98 |
| PC ae C40:0 | 4.54 (1.28) | -0.069 | 1 |  | 0.075 | 1 |  | -0.088 | 1 |  | 0.029 | 1 |  | -0.058 | 1 |  | 0.039 | 1 |
| PC ae C40:1 | 1.69 (0.57) | 0.073 | 1 |  | 0.034 | 1 |  | -0.09 | 1 |  | -0.0032 | 1 |  | -0.1 | 1 |  | -0.041 | 1 |
| PC ae C40:2 | 0.68 (0.21) | 0.01 | 1 |  | -0.038 | 1 |  | -0.16 | 0.57 |  | -0.023 | 1 |  | 0.028 | 1 |  | 0.047 | 1 |
| PC ae C40:3 | 0.79 (0.21) | -0.048 | 1 |  | 0.033 | 1 |  | -0.12 | 1 |  | -0.0044 | 1 |  | 0.034 | 1 |  | 0.017 | 1 |
| PC ae C40:4 | 2.47 (0.65) | -0.069 | 1 |  | -0.00043 | 1 |  | -0.12 | 1 |  | -0.0069 | 1 |  | -0.025 | 1 |  | -0.012 | 1 |
| PC ae C40:5 | 3.81 (0.87) | -0.16 | 1 |  | 0.03 | 1 |  | -0.12 | 1 |  | 0.031 | 1 |  | -0.0025 | 1 |  | 0.031 | 1 |
| PC ae C40:6 | 4.30 (0.98) | -0.17 | 1 |  | 0.024 | 1 |  | -0.094 | 1 |  | 0.0075 | 1 |  | -0.076 | 1 |  | 0.095 | 1 |
| PC ae C42:0 | 0.28 (0.11) | -0.035 | 1 |  | 0.013 | 1 |  | -0.064 | 1 |  | 0.043 | 1 |  | -0.13 | 1 |  | 0.01 | 1 |
| PC ae C42:1 | 0.42 (0.2) | -0.061 | 1 |  | 0.027 | 1 |  | -0.043 | 1 |  | 0.027 | 1 |  | -0.0075 | 1 |  | 0.014 | 1 |
| PC ae C42:2 | 0.55 (0.18) | 0.0028 | 1 |  | 0.025 | 1 |  | -0.11 | 1 |  | -0.0072 | 1 |  | -0.084 | 1 |  | -0.03 | 1 |
| PC ae C42:3 | 0.68 (0.2) | 0.012 | 1 |  | 0.047 | 1 |  | -0.1 | 1 |  | -0.037 | 1 |  | 0.038 | 1 |  | -0.05 | 1 |
| PC ae C42:4 | 0.88 (0.29) | -0.1 | 1 |  | -0.0004 | 1 |  | -0.051 | 1 |  | -0.021 | 1 |  | 0.01 | 1 |  | 0.025 | 1 |
| PC ae C42:5 | 1.51 (0.49) | -0.11 | 1 |  | 0.032 | 1 |  | -0.059 | 1 |  | 0.018 | 1 |  | -0.033 | 1 |  | -0.0075 | 1 |
| PC ae C42:6 | 1.12 (0.33) | -0.083 | 1 |  | 0.034 | 1 |  | -0.092 | 1 |  | 0.036 | 1 |  | -0.079 | 1 |  | -0.035 | 1 |
| SM C35:0 | 0.82 (0.33) | 0.066 | 1 |  | 0.0077 | 1 |  | -0.12 | 1 |  | -0.0042 | 1 |  | 0.2 | 1 |  | -0.032 | 1 |
| SM C35:1 | 3.18 (1.31) | -0.047 | 1 |  | 0.048 | 1 |  | -0.098 | 1 |  | 0.021 | 1 |  | 0.15 | 1 |  | 0.035 | 1 |
| SM C36:0 | 2.25 (0.99) | 0.054 | 1 |  | 0.0027 | 1 |  | -0.077 | 1 |  | -0.0047 | 1 |  | 0.00094 | 1 |  | 0.019 | 1 |
| SM C36:1 | 23.5 (8.08) | -0.071 | 1 |  | 0.039 | 1 |  | -0.11 | 1 |  | 0.0075 | 1 |  | 0.26 | 0.15 |  | 0.11 | 1 |
| SM C36:2 | 10.7 (4.33) | -0.015 | 1 |  | 0.036 | 1 |  | -0.089 | 1 |  | 0.032 | 1 |  | 0.29 | 0.003 |  | 0.12 | 1 |
| SM C37:1 | 2.14 (0.72) | -0.049 | 1 |  | 0.042 | 1 |  | -0.11 | 1 |  | 0.0046 | 1 |  | 0.21 | 1 |  | 0.083 | 1 |
| SM C38:1 | 35.9 (10.6) | -0.049 | 1 |  | 0.084 | 1 |  | -0.061 | 1 |  | 0.037 | 1 |  | 0.24 | 1 |  | 0.075 | 1 |
| SM C38:2 | 19.3 (7.19) | 0.019 | 1 |  | 0.06 | 1 |  | -0.044 | 1 |  | 0.042 | 1 |  | 0.19 | 0.29 |  | 0.027 | 1 |
| SM C38:3 | 0.61 (0.29) | 0.047 | 1 |  | 0.03 | 1 |  | -0.079 | 1 |  | 0.044 | 1 |  | 0.029 | 1 |  | 0.13 | 1 |
| SM C39:1 | 5.34 (1.46) | -0.033 | 1 |  | 0.068 | 1 |  | -0.17 | 0.55 |  | 0.0081 | 1 |  | 0.25 | 1 |  | 0.14 | 1 |
| SM C39:2 | 1.40 (0.44) | 0.048 | 1 |  | 0.078 | 1 |  | -0.12 | 1 |  | 0.035 | 1 |  | 0.099 | 1 |  | 0.12 | 1 |
| SM C40:1 | 26.4 (10.1) | 0.019 | 1 |  | 0.03 | 1 |  | -0.093 | 1 |  | -0.039 | 1 |  | 0.063 | 1 |  | -0.015 | 1 |
| SM C40:2 | 31.6 (6.94) | 0.06 | 1 |  | 0.077 | 1 |  | -0.087 | 1 |  | 0.017 | 1 |  | 0.22 | 1 |  | 0.087 | 1 |
| SM C40:3 | 9.51 (3.41) | 0.059 | 1 |  | -0.024 | 1 |  | -0.095 | 1 |  | 0.026 | 1 |  | 0.03 | 1 |  | 0.096 | 1 |
| SM C40:4 | 6.57 (2.69) | 0.013 | 1 |  | 0.015 | 1 |  | -0.072 | 1 |  | 0.085 | 1 |  | -0.037 | 1 |  | 0.07 | 1 |
| SM C39:5 | 0.57 (0.29) | -0.11 | 1 |  | 0.027 | 1 |  | -0.094 | 1 |  | 0.023 | 1 |  | 0.011 | 1 |  | 0.21 | 0.09 |
| SM C41:1 | 10.9 (2.89) | -0.027 | 1 |  | 0.033 | 1 |  | -0.16 | 0.15 |  | -0.02 | 1 |  | 0.088 | 1 |  | 0.067 | 1 |
| SM C41:2 | 7.97 (1.89) | -0.0076 | 1 |  | 0.062 | 1 |  | -0.15 | 0.32 |  | 0.019 | 1 |  | 0.25 | 1 |  | 0.12 | 1 |
| SM C42:1 | 17.9 (5.68) | -0.0018 | 1 |  | 0.033 | 1 |  | -0.098 | 1 |  | -0.028 | 1 |  | -0.043 | 1 |  | 0.016 | 1 |
| SM C42:2 | 44.7 (11.5) | -0.029 | 1 |  | 0.063 | 1 |  | -0.092 | 1 |  | 0.014 | 1 |  | 0.016 | 1 |  | 0.035 | 1 |
| SM C42:3 | 21.4 (4.81) | -0.017 | 1 |  | 0.057 | 1 |  | -0.085 | 1 |  | 0.022 | 1 |  | 0.15 | 1 |  | 0.15 | 1 |
| SM C41:3 | 1.18 (0.43) | 0.055 | 1 |  | 0.067 | 1 |  | -0.041 | 1 |  | -0.022 | 1 |  | 0.064 | 1 |  | 0.024 | 1 |
| SM C42:4 | 7.29 (1.89) | 0.054 | 1 |  | 0.017 | 1 |  | -0.14 | 1 |  | 0.11 | 1 |  | 0.025 | 1 |  | 0.11 | 1 |
| SM C42:6 | 2.53 (0.91) | -0.16 | 1 |  | 0.027 | 1 |  | -0.048 | 1 |  | 0.037 | 1 |  | 0.0067 | 1 |  | 0.13 | 1 |
| SM C43:1 | 1.33 (0.47) | -0.049 | 1 |  | 0.042 | 1 |  | -0.077 | 1 |  | -0.0052 | 1 |  | 0.00023 | 1 |  | 0.0041 | 1 |
| SM C43:2 | 2.03 (0.73) | -0.058 | 1 |  | 0.032 | 1 |  | -0.11 | 1 |  | -0.0013 | 1 |  | -0.019 | 1 |  | 0.05 | 1 |
| SM C43:0 | 0.56 (0.19) | 0.023 | 1 |  | 0.0092 | 1 |  | -0.067 | 1 |  | -0.032 | 1 |  | -0.0066 | 1 |  | -0.015 | 1 |
| SM C44:2 | 0.33 (0.14) | -0.004 | 1 |  | 0.13 | 0.044 |  | -0.045 | 1 |  | -0.047 | 1 |  | -0.079 | 1 |  | 0.023 | 1 |
| SM C43:3 | 0.51 (0.17) | -0.016 | 1 |  | 0.057 | 1 |  | -0.12 | 1 |  | -0.0024 | 1 |  | -0.026 | 1 |  | 0.053 | 1 |
| SM C44:6 | 1.06 (0.43) | -0.069 | 1 |  | -0.011 | 1 |  | -0.041 | 1 |  | 0.015 | 1 |  | 0.019 | 1 |  | 0.1 | 1 |
| NEFA 10:0 | 1.84 (1.01) | -0.23 | 1 |  | 0.051 | 1 |  | -0.17 | 0.52 |  | -0.0073 | 1 |  | -0.017 | 1 |  | 0.083 | 1 |
| NEFA 12:0 | 6.73 (4.38) | -0.24 | 0.29 |  | 0.0011 | 1 |  | -0.13 | 1 |  | -0.09 | 1 |  | 0.049 | 1 |  | 0.17 | 1 |
| NEFA 12:1 | 1.11 (0.96) | -0.21 | 0.08 |  | 0.042 | 1 |  | -0.15 | 0.024 |  | -0.078 | 1 |  | 0.2 | 1 |  | 0.2 | 0.06 |
| NEFA 14:0 | 11.0 (7.1) | -0.28 | 0.03 |  | 0.03 | 1 |  | -0.15 | 0.26 |  | -0.063 | 1 |  | 0.099 | 1 |  | 0.19 | 0.91 |
| NEFA 14:1 | 1.92 (1.18) | -0.19 | 0.97 |  | 0.05 | 1 |  | -0.16 | 0.06 |  | -0.072 | 1 |  | 0.15 | 1 |  | 0.18 | 0.67 |
| NEFA 15:0 | 2.28 (1.55) | -0.27 | 0.033 |  | 0.022 | 1 |  | -0.15 | 0.19 |  | -0.056 | 1 |  | 0.1 | 1 |  | 0.2 | 0.41 |
| NEFA 15:1 | 0.18 (0.11) | -0.15 | 1 |  | 0.061 | 1 |  | -0.14 | 0.47 |  | -0.077 | 1 |  | 0.12 | 1 |  | 0.14 | 1 |
| NEFA 16:0 | 111.0 (56.7) | -0.33 | 0.003 |  | 0.015 | 1 |  | -0.17 | 0.11 |  | -0.059 | 1 |  | 0.16 | 1 |  | 0.24 | 0.11 |
| NEFA 16:1 | 20.0 (13.1) | -0.35 | 0.001 |  | 0.05 | 1 |  | -0.19 | 0.046 |  | -0.056 | 1 |  | 0.26 | 1 |  | 0.26 | 0.045 |
| NEFA 16:2 | 0.21 (0.15) | -0.15 | 1 |  | 0.014 | 1 |  | -0.084 | 1 |  | -0.028 | 1 |  | 0.12 | 1 |  | 0.15 | 1 |
| NEFA 17:0 | 2.59 (1.5) | -0.25 | 0.06 |  | 0.022 | 1 |  | -0.13 | 0.68 |  | -0.039 | 1 |  | 0.097 | 1 |  | 0.19 | 0.58 |
| NEFA 17:1 | 1.55 (1.04) | -0.29 | 0.015 |  | 0.033 | 1 |  | -0.16 | 0.13 |  | -0.05 | 1 |  | 0.14 | 1 |  | 0.26 | 0.029 |
| NEFA 18:0 | 41.4 (20.8) | -0.26 | 0.049 |  | 0.0016 | 1 |  | -0.12 | 1 |  | -0.044 | 1 |  | 0.063 | 1 |  | 0.14 | 1 |
| NEFA 18:1 | 156.3 (85.4) | -0.36 | 3.7×10^-4^ |  | 0.05 | 1 |  | -0.14 | 0.89 |  | -0.053 | 1 |  | 0.2 | 1 |  | 0.24 | 0.11 |
| NEFA 18:2 | 43.6 (22.2) | -0.35 | 0.002 |  | 0.047 | 1 |  | -0.15 | 0.87 |  | -0.043 | 1 |  | 0.19 | 1 |  | 0.25 | 0.11 |
| NEFA 18:3 | 4.77 (2.9) | -0.22 | 0.63 |  | 0.037 | 1 |  | -0.15 | 0.64 |  | -0.019 | 1 |  | 0.044 | 1 |  | 0.22 | 0.33 |
| NEFA 19:1 | 0.7 (0.42) | -0.29 | 0.006 |  | 0.03 | 1 |  | -0.14 | 0.32 |  | -0.032 | 1 |  | 0.13 | 1 |  | 0.21 | 0.22 |
| NEFA 20:0 | 0.46 (0.28) | -0.25 | 0.23 |  | 0.017 | 1 |  | -0.095 | 1 |  | -0.023 | 1 |  | -0.098 | 1 |  | 0.13 | 1 |
| NEFA 20:2 | 0.97 (0.61) | -0.22 | 0.39 |  | 0.02 | 1 |  | -0.12 | 1 |  | -0.037 | 1 |  | -0.0039 | 1 |  | 0.2 | 0.32 |
| NEFA 20:3 | 1.04 (0.52) | -0.083 | 1 |  | 0.0081 | 1 |  | -0.13 | 1 |  | -0.076 | 1 |  | -0.032 | 1 |  | 0.16 | 1 |
| NEFA 20:4 | 3.29 (1.28) | -0.11 | 1 |  | -0.025 | 1 |  | -0.16 | 0.7 |  | 0.0023 | 1 |  | -0.14 | 1 |  | 0.17 | 1 |
| NEFA 20:5 | 0.29 (0.19) | -0.078 | 1 |  | -0.0057 | 1 |  | -0.17 | 0.58 |  | -0.022 | 1 |  | -0.14 | 1 |  | 0.17 | 1 |
| NEFA 22:4 | 0.51 (0.25) | -0.27 | 0.08 |  | -0.015 | 1 |  | -0.15 | 0.5 |  | -0.018 | 1 |  | 0.062 | 1 |  | 0.31 | 0.002 |
| NEFA 22:5 | 0.92 (0.48) | -0.22 | 0.76 |  | 0.015 | 1 |  | -0.16 | 0.17 |  | -0.034 | 1 |  | 0.048 | 1 |  | 0.23 | 0.21 |
| NEFA 22:6 | 1.85 (0.84) | -0.2 | 1 |  | 0.011 | 1 |  | -0.093 | 1 |  | -0.039 | 1 |  | 0.012 | 1 |  | 0.18 | 1 |
| NEFA 24:0 | 0.15 (0.075) | -0.08 | 1 |  | 0.061 | 1 |  | 0.017 | 1 |  | 0.021 | 1 |  | -0.14 | 1 |  | 0.09 | 1 |
| NEFA 24:2 | 0.04 (0.017) | -0.14 | 1 |  | -0.0097 | 1 |  | -0.088 | 1 |  | 0.0071 | 1 |  | 0.061 | 1 |  | 0.17 | 1 |
| NEFA 24:4 | 0.031 (0.015) | -0.13 | 1 |  | -0.043 | 1 |  | -0.12 | 1 |  | -0.0036 | 1 |  | 0.02 | 1 |  | 0.13 | 1 |
| NEFA 24:5 | 0.034 (0.016) | -0.14 | 1 |  | 0.0096 | 1 |  | -0.075 | 1 |  | -0.06 | 1 |  | 0.11 | 1 |  | 0.19 | 1 |
| NEFA 26:1 | 0.082 (0.043) | 0.013 | 1 |  | 0.026 | 1 |  | 0.014 | 1 |  | -0.023 | 1 |  | 0.073 | 1 |  | 0.13 | 1 |
| Sum NEFA | 431.8 (213) | -0.34 | 0.002 |  | 0.05 | 1 |  | -0.15 | 0.65 |  | -0.081 | 1 |  | 0.12 | 1 |  | 0.18 | 1 |

Abbreviations: Hpro , Hydroxy-Proline, Carn, acylcarnitine; LCA, long-chain acylcarnitines; LPC, lysophosphatidylcholine; PCaa, diacyl-phosphatidylcholine; PCae, acyl-alkyl-phosphatidylcholine; SM, sphingomyeline; NEFA, non-esterified acid
