# Supplementary material for: Metabolomics reveals an entanglement of fasting leptin concentrations with fatty acid oxidation and gluconeogenesis in healthy children
Source: PLoS One. 2017 Aug 17;12(8):e0183185. doi: 10.1371/journal.pone.0183185 (PMC5560563; doi:10.1371/journal.pone.0183185)
Supplement: S2 Table — Variable selection was done by successively eliminating 20% of the least important variables. The mean squared error (MSE) was calculated using 10-fold cross validation and indicates the average of the squares of the prediction errors using the respective number of variables. (DOCX) [file pone.0183185.s003.docx]

**S2 Table.** Variable importance as assessed by the random forests on leptin, adiponectin, and insulin. Variable selection was done by successively eliminating 20% of the least important variables. The mean squared error (MSE) was calculated using 10-fold cross validation and indicates the average of the squares of the prediction errors using the respective number of variables.

|  | **Leptin** | | |  | |  | **Adiponectin** | | | | |  | |  | **Insulin** | | | | |  |
| --- | --- | --- | --- | --- | --- | --- | --- | --- | --- | --- | --- | --- | --- | --- | --- | --- | --- | --- | --- | --- |
| **Metabolites** | **Mean Decreased Accuracy** | **Nb. of variables examined** | **MSE** |  | | **Metabolites** | **Mean Decreased Accuracy** | **Nb. of variables examined** | | **MSE** | |  | | **Metabolites** | **Mean Decreased Accuracy** | **Nb. of variables examined** | | **MSE** | |  |
| Ala | 0.085 | 1 | 116.7 |  | SM C44:2 | | 0.0013 | | 1 | | 11.52 | |  | Ala | 0.14 | | 1 | | 253.1 | |
| Tyr | 0.047 | 2 | 100.1 |  | SM C38:1 | | 0.00085 | | 2 | | 10.31 | |  | Tyr | 0.098 | | 2 | | 220.9 | |
| Val | 0.028 | 3 | 95.14 |  | PC aa C36:5 | | 0.00066 | | 3 | | 9.917 | |  | Carn C2:0 | 0.067 | | 3 | | 214.5 | |
| PC aa C34:4 | 0.023 | 4 | 91 |  | SM C40:2 | | 0.0006 | | 4 | | 9.328 | |  | NEFA 10:0 | 0.057 | | 4 | | 203.8 | |
| NEFA 10:0 | 0.022 | 5 | 86.64 |  | Trp | | 0.00054 | | 5 | | 9.42 | |  | Carn | 0.035 | | 5 | | 195.7 | |
| NEFA 18:0 | 0.019 |  |  |  | Ala | | 0.00046 | |  | |  | |  | NEFA 16:2 | 0.03 | |  | |  | |
| Asp | 0.018 | 7 | 82.28 |  | PC ae C36:0 | | 0.00043 | | 7 | | 9.584 | |  | NEFA 16:1 | 0.028 | | 7 | | 197.2 | |
| PC ae C40:6 | 0.013 |  |  |  | PC ae C34:4 | | 0.00041 | |  | |  | |  | Carn C18:1 | 0.023 | |  | |  | |
| LPC a C14:0 | 0.011 | 9 | 76.35 |  | PC aa C40:1 | | 0.0004 | | 9 | | 9.66 | |  | Carn C3:0 | 0.023 | | 9 | | 196.2 | |
| PC aa C32:3 | 0.011 |  |  |  | PC ae C38:0 | | 0.00039 | |  | |  | |  | NEFA 15:0 | 0.021 | |  | |  | |
| SM C40:2 | 0.01 | 11 | 75.97 |  | Carn C12:1 | | 0.00038 | | 11 | | 9.619 | |  | Carn C12:1 | 0.02 | | 11 | | 195 | |
| NEFA 26:1 | 0.01 |  |  |  | NEFA 18:0 | | 0.00037 | |  | |  | |  | Carn C16:0 | 0.019 | |  | |  | |
| LPC a C22:6 | 0.0094 | 13 | 74.59 |  | PC aa C34:5 | | 0.00037 | | 13 | | 9.791 | |  | NEFA 14:0 | 0.018 | | 13 | | 199 | |
| Glu | 0.0082 |  |  |  | SM C42:1 | | 0.00032 | |  | |  | |  | NEFA 17:1 | 0.016 | |  | |  | |
| Carn C2:0 | 0.008 |  |  |  | PC ae C34:1 | | 0.00032 | |  | |  | |  | PC aa C36:2 | 0.012 | |  | |  | |
| LPC e C18:0 | 0.0078 |  |  |  | SM C38:2 | | 0.00032 | |  | |  | |  | LPC a C14:0 | 0.012 | |  | |  | |
| PC ae C38:3 | 0.0077 | 17 | 71.42 |  | PC ae C40:4 | | 0.00031 | | 17 | | 9.589 | |  | Carn C10:1 | 0.012 | | 17 | | 194.9 | |
| Pro | 0.0076 |  |  |  | NEFA 20:4 | | 0.0003 | |  | |  | |  | Pro | 0.011 | |  | |  | |
| PC aa C38:3 | 0.0067 |  |  |  | PC aa C42:5 | | 0.0003 | |  | |  | |  | Gly | 0.011 | |  | |  | |
| NEFA 18:1 | 0.0062 |  |  |  | SM C40:1 | | 0.0003 | |  | |  | |  | Carn C14:1 | 0.011 | |  | |  | |
| PC aa C36:5 | 0.0061 | 21 | 69.39 |  | PC ae C40:0 | | 0.00029 | | 21 | | 9.541 | |  | NEFA 16:0 | 0.011 | | 21 | | 197.5 | |
| LPC a C20:0 | 0.0058 |  |  |  | NEFA 20:3 | | 0.00028 | |  | |  | |  | PC aa C36:5 | 0.01 | |  | |  | |
| Carn C3:0 | 0.0056 |  |  |  | LPC a C16:0 | | 0.00028 | |  | |  | |  | PC ae C40:0 | 0.01 | |  | |  | |
| PC ae C32:2 | 0.0055 |  |  |  | PC ae C34:2 | | 0.00027 | |  | |  | |  | NEFA 15:1 | 0.0098 | |  | |  | |
| SM C42:3 | 0.0053 |  |  |  | PC aa C42:6 | | 0.00027 | |  | |  | |  | PC ae C40:6 | 0.0097 | |  | |  | |
| PC ae C36:6 | 0.0047 | 26 | 68.97 |  | LPC a C18:1 | | 0.00027 | | 26 | | 9.37 | |  | NEFA 19:1 | 0.009 | | 26 | | 196.7 | |
| PC ae C38:0 | 0.0046 |  |  |  | PC aa C32:3 | | 0.00027 | |  | |  | |  | PC aa C38:2 | 0.009 | |  | |  | |
| PC ae C36:2 | 0.0044 |  |  |  | SM C40:3 | | 0.00026 | |  | |  | |  | PC ae C38:4 | 0.0089 | |  | |  | |
| PC ae C42:6 | 0.0042 |  |  |  | PC ae C36:4 | | 0.00026 | |  | |  | |  | LPC e C18:1 | 0.0085 | |  | |  | |
| PC aa C40:0 | 0.0041 |  |  |  | PC ae C38:4 | | 0.00026 | |  | |  | |  | PC ae C36:2 | 0.0082 | |  | |  | |
| SM C36:2 | 0.004 |  |  |  | PC aa C18:0 | | 0.00025 | |  | |  | |  | NEFA 12:0 | 0.0079 | |  | |  | |
| PC ae C42:3 | 0.0039 |  |  |  | PC aa C42:4 | | 0.00024 | |  | |  | |  | SM C42:3 | 0.0078 | |  | |  | |
| SM C41:2 | 0.0038 | 33 | 68.66 |  | PC aa C34:4 | | 0.00024 | | 33 | | 9.203 | |  | PC ae C40:2 | 0.0075 | | 33 | | 196.3 | |
| His | 0.0036 |  |  |  | PC ae C34:3 | | 0.00024 | |  | |  | |  | NEFA 20:4 | 0.0074 | |  | |  | |
| NEFA 15:0 | 0.0036 |  |  |  | LPC a C20:5 | | 0.00023 | |  | |  | |  | LPC a C18:0 | 0.0073 | |  | |  | |
| NEFA 12:0 | 0.0035 |  |  |  | PC ae C32:2 | | 0.00023 | |  | |  | |  | PC ae C38:3 | 0.0073 | |  | |  | |
| PC ae C40:5 | 0.0035 |  |  |  | LPC e C18:1 | | 0.00023 | |  | |  | |  | PC aa C40:4 | 0.0072 | |  | |  | |
| PC ae C40:0 | 0.0035 |  |  |  | SM C42:2 | | 0.00023 | |  | |  | |  | PC ae C36:1 | 0.0071 | |  | |  | |
| Carn C16:0 | 0.0034 |  |  |  | SM C42:6 | | 0.00023 | |  | |  | |  | SM C41:1 | 0.0069 | |  | |  | |
| PC aa C36:6 | 0.0034 |  |  |  | SM C42:4 | | 0.00022 | |  | |  | |  | PC ae C40:3 | 0.0064 | |  | |  | |
| Ile | 0.0034 | 41 | 68.18 |  | Carn C18:0 | | 0.00022 | | 41 | | 9.148 | |  | LPC a C22:5 | 0.0063 | | 41 | | 196.1 | |
| PC aa C34:5 | 0.0032 |  |  |  | SM C41:2 | | 0.00022 | |  | |  | |  | PC aa C40:6 | 0.0062 | |  | |  | |
| Carn C18:1 | 0.0032 |  |  |  | PC aa C34:2 | | 0.00022 | |  | |  | |  | Thr | 0.0059 | |  | |  | |
| PC aa C36:2 | 0.0032 |  |  |  | LPC a C16:1 | | 0.00022 | |  | |  | |  | PC aa C38:4 | 0.0059 | |  | |  | |
| Carn | 0.0032 |  |  |  | PC aa C38:5 | | 0.00021 | |  | |  | |  | NEFA 20:0 | 0.0059 | |  | |  | |
| PC aa C40:6 | 0.0032 |  |  |  | PC ae C40:1 | | 0.00021 | |  | |  | |  | LPC e C18:0 | 0.0058 | |  | |  | |
| LPC a C22:5 | 0.0032 |  |  |  | PC aa C42:0 | | 0.00021 | |  | |  | |  | NEFA 18:3 | 0.0058 | |  | |  | |
| SM C42:6 | 0.0032 |  |  |  | NEFA 20:2 | | 0.0002 | |  | |  | |  | PC aa C32:2 | 0.0058 | |  | |  | |
| NEFA 16:0 | 0.0031 |  |  |  | PC ae C38:2 | | 0.0002 | |  | |  | |  | SM C35:1 | 0.0057 | |  | |  | |
| PC ae C38:4 | 0.0031 |  |  |  | Tyr | | 0.0002 | |  | |  | |  | PC ae C40:5 | 0.0055 | |  | |  | |
| Cit | 0.0031 | 51 | 68.21 |  | Asp | | 0.00019 | | 51 | | 9.13 | |  | PC ae C36:0 | 0.0054 | | 51 | | 196.4 | |
| PC aa C38:6 | 0.0031 |  |  |  | NEFA 20:0 | | 0.00019 | |  | |  | |  | Asn | 0.0054 | |  | |  | |
| Leu | 0.0031 |  |  |  | PC aa C36:4 | | 0.00019 | |  | |  | |  | Gln | 0.0053 | |  | |  | |
| PC ae C32:0 | 0.003 |  |  |  | NEFA 18:3 | | 0.00019 | |  | |  | |  | SM C40:1 | 0.0051 | |  | |  | |
| PC aa C32:2 | 0.003 |  |  |  | LPC a C22:5 | | 0.00019 | |  | |  | |  | LPC a C18:1 | 0.0051 | |  | |  | |
| PC aa C40:5 | 0.0028 |  |  |  | NEFA 22:4 | | 0.00019 | |  | |  | |  | PC aa C38:5 | 0.005 | |  | |  | |
| Gln | 0.0028 |  |  |  | PC ae C36:2 | | 0.00018 | |  | |  | |  | SM C42:1 | 0.005 | |  | |  | |
| SM C39:2 | 0.0027 |  |  |  | PC ae C36:3 | | 0.00018 | |  | |  | |  | NEFA 24:0 | 0.0049 | |  | |  | |
| PC aa C42:2 | 0.0027 |  |  |  | Carn C16:0 | | 0.00017 | |  | |  | |  | PC ae C42:6 | 0.0049 | |  | |  | |
| Carn C8:1 | 0.0026 |  |  |  | PC ae C42:5 | | 0.00017 | |  | |  | |  | NEFA 17:0 | 0.0049 | |  | |  | |
| Carn C12:1 | 0.0026 |  |  |  | Asn | | 0.00017 | |  | |  | |  | PC ae C38:2 | 0.0047 | |  | |  | |
| NEFA 14:0 | 0.0025 |  |  |  | NEFA 12:1 | | 0.00017 | |  | |  | |  | SM C44:6 | 0.0047 | |  | |  | |
| Asn | 0.0024 |  |  |  | SM C36:1 | | 0.00017 | |  | |  | |  | Cit | 0.0046 | |  | |  | |
| NEFA 20:3 | 0.0024 | 64 | 67.87 |  | SM C43:3 | | 0.00016 | | 64 | | 9.059 | |  | PC aa C32:3 | 0.0046 | | 64 | | 196.4 | |
| PC ae C32:1 | 0.0023 |  |  |  | PC ae C42:1 | | 0.00016 | |  | |  | |  | SM C40:2 | 0.0046 | |  | |  | |
| PC ae C34:4 | 0.0023 |  |  |  | PC aa C34:3 | | 0.00016 | |  | |  | |  | NEFA 26:1 | 0.0045 | |  | |  | |
| LPC a C16:1 | 0.0023 |  |  |  | PC aa C34:1 | | 0.00016 | |  | |  | |  | PC aa C36:6 | 0.0045 | |  | |  | |
| SM C40:3 | 0.0023 |  |  |  | PC aa C36:2 | | 0.00016 | |  | |  | |  | SM C36:0 | 0.0044 | |  | |  | |
| Arg | 0.0022 |  |  |  | PC ae C30:0 | | 0.00015 | |  | |  | |  | PC aa C34:2 | 0.0044 | |  | |  | |
| SM C35:1 | 0.0022 |  |  |  | NEFA 15:1 | | 0.00015 | |  | |  | |  | Asp | 0.0043 | |  | |  | |
| Ser | 0.0021 |  |  |  | PC aa C38:1 | | 0.00015 | |  | |  | |  | SM C43:2 | 0.0042 | |  | |  | |
| NEFA 19:1 | 0.002 |  |  |  | SM C41:3 | | 0.00015 | |  | |  | |  | NEFA 20:5 | 0.004 | |  | |  | |
| PC ae C42:5 | 0.002 |  |  |  | PC ae C40:2 | | 0.00015 | |  | |  | |  | PC ae C32:1 | 0.0039 | |  | |  | |
| PC ae C40:4 | 0.002 |  |  |  | PC ae C40:5 | | 0.00015 | |  | |  | |  | SM C37:1 | 0.0039 | |  | |  | |
| SM C38:3 | 0.002 |  |  |  | PC aa C36:0 | | 0.00015 | |  | |  | |  | PC aa C34:1 | 0.0038 | |  | |  | |
| Cys | 0.002 |  |  |  | PC aa C32:0 | | 0.00014 | |  | |  | |  | PC ae C30:0 | 0.0038 | |  | |  | |
| PC ae C34:1 | 0.002 |  |  |  | PC aa C40:5 | | 0.00014 | |  | |  | |  | SM C36:2 | 0.0037 | |  | |  | |
| PC ae C34:3 | 0.0019 |  |  |  | PC ae C38:5 | | 0.00014 | |  | |  | |  | PC aa C32:1 | 0.0037 | |  | |  | |
| SM C39:5 | 0.0019 |  |  |  | NEFA 18:1 | | 0.00014 | |  | |  | |  | PC ae C36:4 | 0.0037 | |  | |  | |
| PC ae C42:4 | 0.0019 | 80 | 68.25 |  | LPC a C20:3 | | 0.00014 | | 80 | | 9.043 | |  | LPC a C20:2 | 0.0036 | | 80 | | 197 | |
| PC ae C36:1 | 0.0019 |  |  |  | SM C38:3 | | 0.00014 | |  | |  | |  | Ser | 0.0036 | |  | |  | |
| PC aa C38:4 | 0.0018 |  |  |  | LPC a C18:6 | | 0.00014 | |  | |  | |  | PC ae C38:5 | 0.0036 | |  | |  | |
| PC aa C42:6 | 0.0018 |  |  |  | NEFA 24:5 | | 0.00013 | |  | |  | |  | PC ae C32:0 | 0.0036 | |  | |  | |
| PC ae C40:3 | 0.0018 |  |  |  | PC aa C34:0 | | 0.00013 | |  | |  | |  | PC ae C42:0 | 0.0035 | |  | |  | |
| Met | 0.0017 |  |  |  | PC aa C36:3 | | 0.00013 | |  | |  | |  | SM C39:1 | 0.0034 | |  | |  | |
| SM C40:1 | 0.0016 |  |  |  | Arg | | 0.00013 | |  | |  | |  | PC ae C40:1 | 0.0034 | |  | |  | |
| SM C41:3 | 0.0016 |  |  |  | PC aa C30:0 | | 0.00013 | |  | |  | |  | PC aa C38:6 | 0.0034 | |  | |  | |
| NEFA 16:2 | 0.0016 |  |  |  | NEFA 24:2 | | 0.00012 | |  | |  | |  | PC aa C36:3 | 0.0033 | |  | |  | |
| NEFA 22:4 | 0.0016 |  |  |  | PC ae C42:6 | | 0.00012 | |  | |  | |  | PC ae C42:1 | 0.0033 | |  | |  | |
| PC ae C38:6 | 0.0016 |  |  |  | PC aa C40:3 | | 0.00012 | |  | |  | |  | NEFA 24:2 | 0.0033 | |  | |  | |
| SM C38:2 | 0.0014 |  |  |  | NEFA 22:5 | | 0.00012 | |  | |  | |  | PC aa C34:4 | 0.0033 | |  | |  | |
| PC aa C18:1 | 0.0014 |  |  |  | PC aa C32:1 | | 0.00012 | |  | |  | |  | PC ae C34:3 | 0.0032 | |  | |  | |
| PC ae C38:2 | 0.0014 |  |  |  | SM C37:1 | | 0.00012 | |  | |  | |  | PC ae C34:2 | 0.0032 | |  | |  | |
| PC aa C40:1 | 0.0014 |  |  |  | PC ae C42:3 | | 0.00012 | |  | |  | |  | PC aa C42:4 | 0.0032 | |  | |  | |
| PC aa C34:3 | 0.0014 |  |  |  | PC ae C40:6 | | 0.00011 | |  | |  | |  | LPC a C16:1 | 0.0031 | |  | |  | |
| Carn C5:0 | 0.0014 |  |  |  | LPC a C18:2 | | 0.00011 | |  | |  | |  | SM C43:0 | 0.0031 | |  | |  | |
| NEFA 18:3 | 0.0013 |  |  |  | PC ae C42:2 | | 0.00011 | |  | |  | |  | PC ae C42:3 | 0.0031 | |  | |  | |
| NEFA 16:1 | 0.0013 |  |  |  | Carn C18:1 | | 0.00011 | |  | |  | |  | SM C39:2 | 0.0031 | |  | |  | |
| PC aa C42:0 | 0.0013 |  |  |  | SM C40:4 | | 0.00011 | |  | |  | |  | PC aa C36:0 | 0.0029 | |  | |  | |
| SM C42:4 | 0.0013 | 100 | 68.5 |  | PC aa C40:2 | | 0.00011 | | 100 | | 9.045 | |  | NEFA 18:2 | 0.0029 | | 100 | | 197.3 | |
| SM C44:6 | 0.0013 |  |  |  | SM C42:3 | | 0.00011 | |  | |  | |  | Leu | 0.0029 | |  | |  | |
| SM C43:1 | 0.0013 |  |  |  | SM C44:6 | | 0.00011 | |  | |  | |  | Ile | 0.0029 | |  | |  | |
| LPC a C16:0 | 0.0012 |  |  |  | Carn C2:0 | | 0.00011 | |  | |  | |  | Carn C18:0 | 0.0028 | |  | |  | |
| PC aa C36:3 | 0.0012 |  |  |  | PC ae C42:4 | | 0.00011 | |  | |  | |  | PC aa C38:1 | 0.0028 | |  | |  | |
| PC aa C36:0 | 0.0012 |  |  |  | Lys | | 0.0001 | |  | |  | |  | PC ae C42:2 | 0.0028 | |  | |  | |
| Carn C10:1 | 0.0012 |  |  |  | LPC a C20:0 | | 0.0001 | |  | |  | |  | LPC a C20:0 | 0.0027 | |  | |  | |
| SM C44:2 | 0.0012 |  |  |  | NEFA 16:2 | | 0.0001 | |  | |  | |  | Orn | 0.0027 | |  | |  | |
| NEFA 20:0 | 0.0012 |  |  |  | PC ae C32:0 | | 0.000099 | |  | |  | |  | NEFA 12:1 | 0.0027 | |  | |  | |
| NEFA 17:1 | 0.0012 |  |  |  | NEFA 18:2 | | 0.000098 | |  | |  | |  | LPC a C16:0 | 0.0027 | |  | |  | |
| Carn C18:0 | 0.0012 |  |  |  | PC aa C32:2 | | 0.000093 | |  | |  | |  | PC aa C34:3 | 0.0027 | |  | |  | |
| PC ae C40:1 | 0.0012 |  |  |  | Ile | | 0.000091 | |  | |  | |  | SM C36:1 | 0.0027 | |  | |  | |
| PC aa C18:0 | 0.0011 |  |  |  | SM C35:0 | | 0.00009 | |  | |  | |  | PC aa C38:0 | 0.0026 | |  | |  | |
| PC ae C42:2 | 0.0011 |  |  |  | NEFA 17:1 | | 0.000089 | |  | |  | |  | SM C42:4 | 0.0025 | |  | |  | |
| PC aa C38:0 | 0.0011 |  |  |  | PC aa C43:6 | | 0.000089 | |  | |  | |  | Carn C8:1 | 0.0025 | |  | |  | |
| Phe | 0.0011 |  |  |  | NEFA 22:6 | | 0.000089 | |  | |  | |  | PC aa C38:3 | 0.0024 | |  | |  | |
| SM C36:0 | 0.0011 |  |  |  | PC aa C38:0 | | 0.000088 | |  | |  | |  | NEFA 20:3 | 0.0024 | |  | |  | |
| PC aa C42:5 | 0.0011 |  |  |  | PC aa C40:4 | | 0.000086 | |  | |  | |  | PC aa C40:5 | 0.0023 | |  | |  | |
| LPC a C20:1 | 0.0011 |  |  |  | SM C41:1 | | 0.000086 | |  | |  | |  | Glu | 0.0022 | |  | |  | |
| PC ae C34:2 | 0.0011 |  |  |  | PC ae C32:1 | | 0.000086 | |  | |  | |  | NEFA 18:1 | 0.0022 | |  | |  | |
| SM C42:1 | 0.001 |  |  |  | LPC a C22:4 | | 0.000086 | |  | |  | |  | PC aa C36:1 | 0.0022 | |  | |  | |
| SM C38:1 | 0.001 |  |  |  | NEFA 16:0 | | 0.000085 | |  | |  | |  | NEFA 14:1 | 0.0022 | |  | |  | |
| SM C42:2 | 0.001 |  |  |  | SM C39:1 | | 0.000085 | |  | |  | |  | NEFA 20:2 | 0.0021 | |  | |  | |
| PC aa C38:5 | 0.001 |  |  |  | PC ae C34:0 | | 0.000084 | |  | |  | |  | LPC a C22:4 | 0.002 | |  | |  | |
| SM C39:1 | 0.001 |  |  |  | LPC a C18:0 | | 0.000083 | |  | |  | |  | PC ae C32:2 | 0.002 | |  | |  | |
| Carn C14:1 | 0.001 | 125 | 69.64 |  | SM C35:1 | | 0.00008 | | 125 | | 9.034 | |  | PC aa C30:0 | 0.002 | | 125 | | 197.1 | |
| PC ae C42:0 | 0.00096 |  |  |  | PC aa C18:1 | | 0.00008 | |  | |  | |  | PC ae C40:4 | 0.002 | |  | |  | |
| PC aa C38:2 | 0.00095 |  |  |  | SM C43:2 | | 0.000079 | |  | |  | |  | PC aa C42:0 | 0.0019 | |  | |  | |
| PC aa C38:1 | 0.00095 |  |  |  | PC ae C38:6 | | 0.000079 | |  | |  | |  | SM C38:1 | 0.0019 | |  | |  | |
| Thr | 0.00094 |  |  |  | PC aa C42:1 | | 0.000077 | |  | |  | |  | PC ae C38:0 | 0.0019 | |  | |  | |
| LPC a C20:2 | 0.00093 |  |  |  | SM C36:2 | | 0.000075 | |  | |  | |  | PC ae C36:5 | 0.0019 | |  | |  | |
| LPC a C20:5 | 0.00092 |  |  |  | NEFA 24:4 | | 0.000075 | |  | |  | |  | SM C43:1 | 0.0018 | |  | |  | |
| PC aa C30:2 | 0.0009 |  |  |  | Leu | | 0.000074 | |  | |  | |  | Met | 0.0018 | |  | |  | |
| LPC e C18:1 | 0.00087 |  |  |  | Carn C14:1 | | 0.000073 | |  | |  | |  | SM C42:6 | 0.0018 | |  | |  | |
| SM C35:0 | 0.00086 |  |  |  | NEFA 12:0 | | 0.000071 | |  | |  | |  | PC aa C40:3 | 0.0018 | |  | |  | |
| SM C37:1 | 0.00083 |  |  |  | NEFA 19:1 | | 0.000069 | |  | |  | |  | SM C42:2 | 0.0017 | |  | |  | |
| NEFA 24:0 | 0.00082 |  |  |  | LPC e C16:1 | | 0.000069 | |  | |  | |  | LPC a C22:6 | 0.0017 | |  | |  | |
| PC aa C34:6 | 0.0008 |  |  |  | Ser | | 0.000069 | |  | |  | |  | LPC e C16:0 | 0.0016 | |  | |  | |
| PC aa C30:0 | 0.00079 |  |  |  | PC ae C42:0 | | 0.000069 | |  | |  | |  | PC aa C30:2 | 0.0016 | |  | |  | |
| NEFA 24:2 | 0.00079 |  |  |  | NEFA 14:0 | | 0.000066 | |  | |  | |  | PC aa C32:0 | 0.0015 | |  | |  | |
| SM C41:1 | 0.00076 |  |  |  | LPC a C20:1 | | 0.000065 | |  | |  | |  | LPC a C18:2 | 0.0015 | |  | |  | |
| NEFA 18:2 | 0.00076 |  |  |  | PC ae C36:5 | | 0.000065 | |  | |  | |  | SM C44:2 | 0.0015 | |  | |  | |
| Gly | 0.00075 |  |  |  | Carn C10:1 | | 0.000061 | |  | |  | |  | PC ae C34:4 | 0.0015 | |  | |  | |
| PC aa C32:1 | 0.00075 |  |  |  | NEFA 17:0 | | 0.00006 | |  | |  | |  | PC ae C34:0 | 0.0015 | |  | |  | |
| PC aa C36:1 | 0.00074 |  |  |  | Orn | | 0.000058 | |  | |  | |  | NEFA 24:5 | 0.0015 | |  | |  | |
| PC aa C36:4 | 0.00073 |  |  |  | PC aa C40:0 | | 0.000055 | |  | |  | |  | LPC a C20:5 | 0.0014 | |  | |  | |
| PC aa C43:6 | 0.00072 |  |  |  | PC ae C40:3 | | 0.00005 | |  | |  | |  | His | 0.0014 | |  | |  | |
| PC ae C36:5 | 0.00072 |  |  |  | PC aa C38:4 | | 0.000048 | |  | |  | |  | NEFA 18:0 | 0.0014 | |  | |  | |
| PC ae C40:2 | 0.00072 |  |  |  | LPC a C20:4 | | 0.000045 | |  | |  | |  | PC aa C18:1 | 0.0014 | |  | |  | |
| LPC a C20:3 | 0.00071 |  |  |  | SM C43:1 | | 0.000045 | |  | |  | |  | PC aa C34:0 | 0.0014 | |  | |  | |
| NEFA 12:1 | 0.0007 |  |  |  | LPC e C16:0 | | 0.000042 | |  | |  | |  | SM C41:2 | 0.0014 | |  | |  | |
| SM C43:2 | 0.0007 |  |  |  | PC aa C38:6 | | 0.000041 | |  | |  | |  | PC ae C36:3 | 0.0013 | |  | |  | |
| SM C36:1 | 0.00067 |  |  |  | PC aa C34:6 | | 0.000041 | |  | |  | |  | SM C43:3 | 0.0013 | |  | |  | |
| PC aa C34:2 | 0.00066 |  |  |  | PC ae C36:1 | | 0.000039 | |  | |  | |  | SM C40:4 | 0.0013 | |  | |  | |
| NEFA 20:5 | 0.00066 |  |  |  | Glu | | 0.000035 | |  | |  | |  | PC aa C42:5 | 0.0013 | |  | |  | |
| NEFA 22:6 | 0.00063 |  |  |  | LPC e C18:0 | | 0.000035 | |  | |  | |  | SM C41:3 | 0.0012 | |  | |  | |
| Trp | 0.00062 | 156 | 70.22 |  | LPC a C22:6 | | 0.000035 | | 156 | | 9.006 | |  | PC aa C34:5 | 0.0012 | | 156 | | 196.8 | |
| PC ae C34:0 | 0.00058 |  |  |  | NEFA 24:0 | | 0.000031 | |  | |  | |  | NEFA 24:4 | 0.0012 | |  | |  | |
| LPC e C16:0 | 0.00058 |  |  |  | PC ae C38:3 | | 0.00003 | |  | |  | |  | SM C39:5 | 0.0012 | |  | |  | |
| PC aa C40:2 | 0.00057 |  |  |  | PC aa C38:2 | | 0.00003 | |  | |  | |  | NEFA 22:4 | 0.0012 | |  | |  | |
| Lys | 0.00055 |  |  |  | Gly | | 0.000028 | |  | |  | |  | SM C38:2 | 0.0011 | |  | |  | |
| NEFA 24:4 | 0.00055 |  |  |  | Carn C3:0 | | 0.000028 | |  | |  | |  | SM C40:3 | 0.0011 | |  | |  | |
| PC aa C42:1 | 0.00051 |  |  |  | NEFA 15:0 | | 0.00002 | |  | |  | |  | PC ae C42:5 | 0.0011 | |  | |  | |
| LPC a C18:0 | 0.00051 |  |  |  | Met | | 0.000016 | |  | |  | |  | LPC a C18:3 | 0.0011 | |  | |  | |
| LPC a C18:1 | 0.00051 |  |  |  | PC aa C36:1 | | 0.000013 | |  | |  | |  | PC aa C36:4 | 0.00099 | |  | |  | |
| LPC a C18:6 | 0.00049 |  |  |  | Cit | | 0.0000058 | |  | |  | |  | LPC a C20:3 | 0.00099 | |  | |  | |
| NEFA 22:5 | 0.00048 |  |  |  | NEFA 10:0 | | 0.0000048 | |  | |  | |  | PC aa C43:6 | 0.00096 | |  | |  | |
| PC ae C30:0 | 0.00048 |  |  |  | NEFA 16:1 | | 0.0000047 | |  | |  | |  | Cys | 0.00093 | |  | |  | |
| PC aa C34:0 | 0.00045 |  |  |  | PC aa C36:6 | | 0.0000041 | |  | |  | |  | PC aa C40:2 | 0.0009 | |  | |  | |
| PC aa C40:3 | 0.00044 |  |  |  | PC aa C38:3 | | 0.0000022 | |  | |  | |  | PC ae C34:1 | 0.0009 | |  | |  | |
| SM C40:4 | 0.00044 |  |  |  | His | | -0.00000046 | |  | |  | |  | PC ae C36:6 | 0.00086 | |  | |  | |
| Hpro | 0.00044 |  |  |  | SM C39:5 | | -0.0000011 | |  | |  | |  | PC ae C42:4 | 0.00084 | |  | |  | |
| PC ae C38:5 | 0.00044 |  |  |  | PC ae C36:6 | | -0.0000029 | |  | |  | |  | Arg | 0.00083 | |  | |  | |
| PC ae C36:0 | 0.00043 |  |  |  | SM C36:0 | | -0.0000047 | |  | |  | |  | Carn C5:0 | 0.0008 | |  | |  | |
| LPC a C22:4 | 0.00043 |  |  |  | PC aa C42:2 | | -0.0000054 | |  | |  | |  | Trp | 0.00073 | |  | |  | |
| PC ae C36:3 | 0.00042 |  |  |  | SM C43:0 | | -0.0000094 | |  | |  | |  | NEFA 22:6 | 0.00071 | |  | |  | |
| PC aa C32:0 | 0.00041 |  |  |  | LPC a C14:0 | | -0.000011 | |  | |  | |  | PC aa C40:0 | 0.00066 | |  | |  | |
| Orn | 0.0004 |  |  |  | Gln | | -0.000014 | |  | |  | |  | Val | 0.00056 | |  | |  | |
| NEFA 14:1 | 0.00039 |  |  |  | SM C39:2 | | -0.000017 | |  | |  | |  | PC aa C42:6 | 0.00056 | |  | |  | |
| PC aa C34:1 | 0.00038 |  |  |  | PC aa C40:6 | | -0.000019 | |  | |  | |  | PC ae C38:6 | 0.00051 | |  | |  | |
| LPC a C18:2 | 0.00036 |  |  |  | Hpro | | -0.000023 | |  | |  | |  | LPC a C20:4 | 0.00045 | |  | |  | |
| NEFA 20:4 | 0.00034 |  |  |  | Pro | | -0.000027 | |  | |  | |  | SM C35:0 | 0.00037 | |  | |  | |
| SM C43:0 | 0.00032 |  |  |  | Thr | | -0.000029 | |  | |  | |  | PC aa C40:1 | 0.00035 | |  | |  | |
| LPC a C18:3 | 0.00025 |  |  |  | Carn C4:0 | | -0.00003 | |  | |  | |  | PC aa C42:1 | 0.00026 | |  | |  | |
| LPC a C20:4 | 0.00024 |  |  |  | Phe | | -0.000031 | |  | |  | |  | PC aa C18:0 | -0.00013 | |  | |  | |
| NEFA 24:5 | 0.00018 |  |  |  | Val | | -0.000033 | |  | |  | |  | Phe | -0.00021 | |  | |  | |
| NEFA 17:0 | 0.00015 |  |  |  | NEFA 26:1 | | -0.000046 | |  | |  | |  | Hpro | -0.00029 | |  | |  | |
| LPC e C16:1 | 0.00013 |  |  |  | PC aa C30:2 | | -0.000053 | |  | |  | |  | Carn C4:0 | -0.0003 | |  | |  | |
| PC ae C42:1 | 0.000098 |  |  |  | Carn C5:0 | | -0.000056 | |  | |  | |  | SM C38:3 | -0.00032 | |  | |  | |
| SM C43:3 | 0.000023 |  |  |  | Carn | | -0.000057 | |  | |  | |  | LPC a C18:6 | -0.00039 | |  | |  | |
| PC ae C36:4 | 0.0000061 |  |  |  | LPC a C18:3 | | -0.000057 | |  | |  | |  | PC aa C34:6 | -0.00044 | |  | |  | |
| NEFA 15:1 | 0.0000044 |  |  |  | NEFA 14:1 | | -0.000066 | |  | |  | |  | LPC a C20:1 | -0.00046 | |  | |  | |
| PC aa C42:4 | 0.00000093 |  |  |  | NEFA 20:5 | | -0.000077 | |  | |  | |  | PC aa C42:2 | -0.00083 | |  | |  | |
| PC aa C40:4 | -0.000062 |  |  |  | Carn C8:1 | | -0.00017 | |  | |  | |  | NEFA 22:5 | -0.00084 | |  | |  | |
| NEFA 20:2 | -0.0002 |  |  |  | Cys | | -0.00022 | |  | |  | |  | LPC e C16:1 | -0.00091 | |  | |  | |
| Carn C4:0 | -0.00053 | 195 | 71.02 |  | LPC a C20:2 | | -0.00027 | | 195 | | 8.982 | |  | Lys | -0.002 | | 195 | | 196.7 | |
