# Supplementary material for: Metabolomics reveals an entanglement of fasting leptin concentrations with fatty acid oxidation and gluconeogenesis in healthy children
Source: PLoS One. 2017 Aug 17;12(8):e0183185. doi: 10.1371/journal.pone.0183185 (PMC5560563; doi:10.1371/journal.pone.0183185)
Supplement: S3 Table — The standardized regression coefficient β and the Bonferroni corrected P value [PBF] are given for each interaction effect. CPT-1 reflects the acylcarnitine ratio (C16+C18)/C0; CPT-2 reflects the acylcarnitine ratio (C2/(C16+C18)). (DOCX) [file pone.0183185.s004.docx]

**S3 Table.** Results of the linear mixed models (LMM) regressing each metabolite on fasting leptin, fasting adiponectin, fasting insulin, age, sex, BMI, and the respective interaction terms (added one by one), with a random intercept for batch number. The standardized regression coefficient β and the Bonferroni corrected P value [*P*_BF_] are given for each interaction effect. CPT-1 reflects the acylcarnitine ratio (C16+C18)/C0; CPT-2 reflects the acylcarnitine ratio (C2/(C16+C18)).

| **Metabolite (Outcome)** | **Leptin × Insulin** | |  | **Leptin × BMI** | |  | **Insulin × BMI** | |  | **Leptin × sex** | |  | **Insulin × sex** | |
| --- | --- | --- | --- | --- | --- | --- | --- | --- | --- | --- | --- | --- | --- | --- |
|  | β | *P*_BF_ |  | β | *P*_BF_ |  | β | *P*_BF_ |  | β | *P*_BF_ |  | β | *P*_BF_ |
| Ala | -0.023 | 1 |  | 0.022 | 1 |  | 0.027 | 1 |  | 0.004 | 1 |  | -0.091 | 1 |
| Arg | -0.091 | 1 |  | -0.061 | 1 |  | -0.013 | 1 |  | -0.16 | 1 |  | 0.014 | 1 |
| Asn | -0.068 | 1 |  | -0.067 | 1 |  | -0.048 | 1 |  | 0.052 | 1 |  | 0.058 | 1 |
| Asp | 0.025 | 1 |  | 0.038 | 1 |  | 0.036 | 1 |  | -0.049 | 1 |  | 0.078 | 1 |
| Cit | -0.027 | 1 |  | -0.06 | 1 |  | -0.0028 | 1 |  | -0.11 | 1 |  | -0.065 | 1 |
| Cys | 0.0057 | 1 |  | -0.0083 | 1 |  | 0.0073 | 1 |  | 0.0039 | 1 |  | 0.03 | 1 |
| Gln | -0.086 | 1 |  | -0.086 | 1 |  | -0.063 | 1 |  | 0.011 | 1 |  | 0.088 | 1 |
| Glu | 0.024 | 1 |  | 0.042 | 1 |  | 0.046 | 1 |  | -0.07 | 1 |  | -0.039 | 1 |
| Gly | -0.081 | 1 |  | -0.085 | 1 |  | -0.054 | 1 |  | -0.02 | 1 |  | 0.022 | 1 |
| His | -0.02 | 1 |  | -0.033 | 1 |  | -0.022 | 1 |  | 0.1 | 1 |  | 0.076 | 1 |
| Hpro | 0.028 | 1 |  | -0.069 | 1 |  | 0.00066 | 1 |  | -0.16 | 1 |  | 0.078 | 1 |
| Ile | 0.046 | 1 |  | 0.025 | 1 |  | 0.022 | 1 |  | 0.039 | 1 |  | 0.058 | 1 |
| Leu | 0.1 | 1 |  | 0.024 | 1 |  | 0.019 | 1 |  | 0.023 | 1 |  | 0.16 | 1 |
| Lys | -0.068 | 1 |  | -0.022 | 1 |  | -0.041 | 1 |  | 0.07 | 1 |  | 0.065 | 1 |
| Met | -0.073 | 1 |  | -0.065 | 1 |  | -0.053 | 1 |  | 0.056 | 1 |  | 0.075 | 1 |
| Orn | -0.062 | 1 |  | -0.043 | 1 |  | -0.094 | 1 |  | 0.035 | 1 |  | 0.11 | 1 |
| Phe | -0.042 | 1 |  | -0.05 | 1 |  | -0.043 | 1 |  | 0.059 | 1 |  | 0.13 | 1 |
| Pro | 0.015 | 1 |  | -0.031 | 1 |  | -0.019 | 1 |  | 0.039 | 1 |  | -0.024 | 1 |
| Ser | -0.07 | 1 |  | -0.082 | 1 |  | -0.068 | 1 |  | -0.027 | 1 |  | 0.057 | 1 |
| Thr | -0.073 | 1 |  | -0.12 | 1 |  | -0.059 | 1 |  | -0.023 | 1 |  | 0.058 | 1 |
| Trp | -0.067 | 1 |  | -0.058 | 1 |  | -0.04 | 1 |  | 0.013 | 1 |  | 0.066 | 1 |
| Tyr | -0.0054 | 1 |  | 0.013 | 1 |  | 0.0036 | 1 |  | -0.024 | 1 |  | -0.047 | 1 |
| Val | 0.07 | 1 |  | 0.02 | 1 |  | -0.02 | 1 |  | 0.053 | 1 |  | 0.19 | 1 |
| Glucose | -0.21 | 6.2×10^-5^ |  | -0.097 | 1 |  | -0.15 | 0.019 |  | -0.059 | 1 |  | -0.21 | 1 |
| Carn | -0.031 | 1 |  | -0.048 | 1 |  | -0.018 | 1 |  | -0.094 | 1 |  | -0.0042 | 1 |
| Carn C2:0 | 0.076 | 1 |  | 0.0048 | 1 |  | 0.027 | 1 |  | -0.012 | 1 |  | 0.019 | 1 |
| Carn C3:0 | 0.067 | 1 |  | -0.02 | 1 |  | 0.035 | 1 |  | -0.045 | 1 |  | 0.17 | 1 |
| Carn C4:0 | 0.082 | 1 |  | 0.042 | 1 |  | 0.021 | 1 |  | -0.12 | 1 |  | 0.15 | 1 |
| Carn C5:0 | 0.078 | 1 |  | 0.0016 | 1 |  | 0.043 | 1 |  | 0.11 | 1 |  | 0.07 | 1 |
| Carn C8:1 | 0.0023 | 1 |  | -0.01 | 1 |  | -0.038 | 1 |  | -0.071 | 1 |  | 0.053 | 1 |
| Carn C10:1 | -0.078 | 1 |  | -0.1 | 1 |  | -0.073 | 1 |  | -0.052 | 1 |  | 0.071 | 1 |
| Carn C12:1 | -0.027 | 1 |  | -0.056 | 1 |  | -0.053 | 1 |  | -0.084 | 1 |  | 0.012 | 1 |
| Carn C14:1 | 0.037 | 1 |  | -0.029 | 1 |  | -0.025 | 1 |  | 0.0074 | 1 |  | 0.1 | 1 |
| Carn C16:0 | 0.031 | 1 |  | -0.0061 | 1 |  | -0.036 | 1 |  | 0.049 | 1 |  | 0.11 | 1 |
| Carn C18:0 | 0.082 | 1 |  | 0.035 | 1 |  | 0.073 | 1 |  | -0.14 | 1 |  | 0.027 | 1 |
| Carn C18:1 | 0.046 | 1 |  | -0.0098 | 1 |  | 0.0031 | 1 |  | -0.049 | 1 |  | -0.012 | 1 |
| Sum LCA | 0.04 | 1 |  | -0.017 | 1 |  | -0.0079 | 1 |  | -0.044 | 1 |  | 0.061 | 1 |
| CPT-1 | 0.072 | 1 |  | 0.039 | 1 |  | 0.023 | 1 |  | 0.016 | 1 |  | 0.071 | 1 |
| CPT-2 | 0.034 | 1 |  | 0.011 | 1 |  | 0.044 | 1 |  | 0.023 | 1 |  | -0.024 | 1 |
| LPC a C14:0 | -0.0066 | 1 |  | 0.016 | 1 |  | 0.043 | 1 |  | -0.043 | 1 |  | -0.035 | 1 |
| LPC a C16:0 | -0.053 | 1 |  | -0.016 | 1 |  | -0.026 | 1 |  | -0.11 | 1 |  | -0.05 | 1 |
| LPC a C16:1 | -0.011 | 1 |  | -0.0067 | 1 |  | 0.0018 | 1 |  | -0.17 | 1 |  | -0.078 | 1 |
| LPC a C18:0 | -0.032 | 1 |  | -0.02 | 1 |  | -0.022 | 1 |  | -0.098 | 1 |  | -0.023 | 1 |
| LPC a C18:1 | -0.053 | 1 |  | -0.05 | 1 |  | -0.056 | 1 |  | -0.17 | 1 |  | -0.047 | 1 |
| LPC a C18:2 | -0.023 | 1 |  | -0.061 | 1 |  | -0.054 | 1 |  | -0.058 | 1 |  | 0.047 | 1 |
| LPC a C18:3 | 0.0046 | 1 |  | -0.0049 | 1 |  | -0.034 | 1 |  | -0.24 | 1 |  | -0.053 | 1 |
| LPC a C18:6 | -0.047 | 1 |  | -0.1 | 1 |  | -0.04 | 1 |  | -0.19 | 1 |  | -0.073 | 1 |
| LPC a C20:0 | -0.025 | 1 |  | -0.076 | 1 |  | -0.029 | 1 |  | -0.1 | 1 |  | -0.046 | 1 |
| LPC a C20:1 | -0.048 | 1 |  | -0.068 | 1 |  | -0.029 | 1 |  | -0.22 | 1 |  | -0.14 | 1 |
| LPC a C20:2 | -0.018 | 1 |  | -0.06 | 1 |  | -0.029 | 1 |  | -0.076 | 1 |  | 0.03 | 1 |
| LPC a C20:3 | -0.019 | 1 |  | -0.032 | 1 |  | -0.042 | 1 |  | -0.089 | 1 |  | 0.0094 | 1 |
| LPC a C20:4 | -0.042 | 1 |  | -0.053 | 1 |  | -0.073 | 1 |  | -0.12 | 1 |  | 0.0053 | 1 |
| LPC a C20:5 | 0.027 | 1 |  | 0.048 | 1 |  | -0.0056 | 1 |  | 0.059 | 1 |  | -0.0039 | 1 |
| LPC a C22:4 | 0.0059 | 1 |  | -0.0037 | 1 |  | 0.0069 | 1 |  | -0.087 | 1 |  | -0.024 | 1 |
| LPC a C22:5 | 0.038 | 1 |  | -0.016 | 1 |  | -0.0063 | 1 |  | -0.013 | 1 |  | 0.033 | 1 |
| LPC a C22:6 | -0.084 | 1 |  | -0.037 | 1 |  | -0.072 | 1 |  | -0.061 | 1 |  | -0.036 | 1 |
| LPC e C16:0 | 0.0017 | 1 |  | -0.006 | 1 |  | 0.002 | 1 |  | 0.055 | 1 |  | -0.018 | 1 |
| LPC e C16:1 | 0.028 | 1 |  | -0.069 | 1 |  | -0.035 | 1 |  | -0.11 | 1 |  | -0.0084 | 1 |
| LPC e C18:0 | -0.027 | 1 |  | -0.012 | 1 |  | -0.039 | 1 |  | 0.043 | 1 |  | 0.0039 | 1 |
| LPC e C18:1 | 0.023 | 1 |  | -0.025 | 1 |  | 0.025 | 1 |  | -0.021 | 1 |  | 0.012 | 1 |
| PC aa C18:0 | 0.039 | 1 |  | -0.018 | 1 |  | 0.033 | 1 |  | -0.012 | 1 |  | -0.12 | 1 |
| PC aa C18:1 | 0.049 | 1 |  | 0.021 | 1 |  | 0.023 | 1 |  | -0.028 | 1 |  | 0.055 | 1 |
| PC aa C30:0 | 0.077 | 1 |  | 0.038 | 1 |  | 0.07 | 1 |  | -0.045 | 1 |  | 0.00011 | 1 |
| PC aa C30:2 | -0.018 | 1 |  | -0.046 | 1 |  | -0.042 | 1 |  | -0.079 | 1 |  | -0.073 | 1 |
| PC aa C32:0 | 0.079 | 1 |  | 0.047 | 1 |  | 0.036 | 1 |  | -0.12 | 1 |  | -0.0026 | 1 |
| PC aa C32:1 | 0.092 | 1 |  | 0.064 | 1 |  | 0.082 | 1 |  | -0.084 | 1 |  | 0.027 | 1 |
| PC aa C32:2 | 0.025 | 1 |  | -0.016 | 1 |  | 0.027 | 1 |  | 0.026 | 1 |  | 0.058 | 1 |
| PC aa C32:3 | 0.057 | 1 |  | -0.000079 | 1 |  | 0.0047 | 1 |  | -0.079 | 1 |  | 0.051 | 1 |
| PC aa C34:0 | 0.055 | 1 |  | -0.0051 | 1 |  | 0.039 | 1 |  | -0.043 | 1 |  | 0.0065 | 1 |
| PC aa C34:1 | 0.069 | 1 |  | 0.036 | 1 |  | 0.042 | 1 |  | -0.13 | 1 |  | 0.11 | 1 |
| PC aa C34:2 | 0.069 | 1 |  | 0.016 | 1 |  | 0.025 | 1 |  | 0.023 | 1 |  | 0.18 | 1 |
| PC aa C34:3 | 0.058 | 1 |  | 0.028 | 1 |  | 0.026 | 1 |  | -0.11 | 1 |  | 0.068 | 1 |
| PC aa C34:4 | 0.044 | 1 |  | -0.0041 | 1 |  | 0.03 | 1 |  | -0.066 | 1 |  | 0.049 | 1 |
| PC aa C34:5 | 0.074 | 1 |  | 0.032 | 1 |  | 0.028 | 1 |  | 0.047 | 1 |  | 0.0052 | 1 |
| PC aa C34:6 | 0.022 | 1 |  | -0.032 | 1 |  | -0.0074 | 1 |  | 0.12 | 1 |  | 0.11 | 1 |
| PC aa C36:0 | 0.083 | 1 |  | -0.0055 | 1 |  | 0.044 | 1 |  | 0.022 | 1 |  | -0.028 | 1 |
| PC aa C36:1 | 0.11 | 1 |  | 0.03 | 1 |  | 0.065 | 1 |  | -0.095 | 1 |  | 0.076 | 1 |
| PC aa C36:2 | 0.075 | 1 |  | 0.0089 | 1 |  | 0.021 | 1 |  | 0.0082 | 1 |  | 0.18 | 1 |
| PC aa C36:3 | 0.056 | 1 |  | 0.022 | 1 |  | 0.017 | 1 |  | -0.017 | 1 |  | 0.17 | 1 |
| PC aa C36:4 | 0.072 | 1 |  | -0.00043 | 1 |  | 0.018 | 1 |  | -0.092 | 1 |  | 0.15 | 1 |
| PC aa C36:5 | 0.064 | 1 |  | 0.064 | 1 |  | 0.026 | 1 |  | 0.041 | 1 |  | 0.048 | 1 |
| PC aa C36:6 | 0.013 | 1 |  | -0.0027 | 1 |  | 0.0033 | 1 |  | 0.051 | 1 |  | 0.021 | 1 |
| PC aa C38:0 | -0.065 | 1 |  | -0.066 | 1 |  | -0.091 | 1 |  | 0.095 | 1 |  | 0.1 | 1 |
| PC aa C38:1 | 0.053 | 1 |  | 0.021 | 1 |  | 0.03 | 1 |  | -0.017 | 1 |  | 0.062 | 1 |
| PC aa C38:2 | 0.081 | 1 |  | 0.0049 | 1 |  | 0.054 | 1 |  | -0.0036 | 1 |  | 0.094 | 1 |
| PC aa C38:3 | 0.077 | 1 |  | 0.039 | 1 |  | 0.036 | 1 |  | -0.013 | 1 |  | 0.12 | 1 |
| PC aa C38:4 | 0.072 | 1 |  | 0.003 | 1 |  | 0.011 | 1 |  | -0.075 | 1 |  | 0.11 | 1 |
| PC aa C38:5 | 0.063 | 1 |  | 0.024 | 1 |  | 0.0088 | 1 |  | -0.035 | 1 |  | 0.1 | 1 |
| PC aa C38:6 | -0.035 | 1 |  | -0.014 | 1 |  | -0.038 | 1 |  | 0.047 | 1 |  | 0.05 | 1 |
| PC aa C40:0 | 0.007 | 1 |  | -0.000095 | 1 |  | -0.017 | 1 |  | 0.034 | 1 |  | 0.13 | 1 |
| PC aa C40:1 | -0.047 | 1 |  | -0.045 | 1 |  | -0.057 | 1 |  | -0.027 | 1 |  | 0.0098 | 1 |
| PC aa C40:2 | -0.0045 | 1 |  | 0.029 | 1 |  | -0.016 | 1 |  | 0.12 | 1 |  | 0.14 | 1 |
| PC aa C40:3 | -0.013 | 1 |  | -0.031 | 1 |  | -0.035 | 1 |  | -0.1 | 1 |  | -0.061 | 1 |
| PC aa C40:4 | 0.092 | 1 |  | 0.03 | 1 |  | 0.043 | 1 |  | -0.065 | 1 |  | 0.1 | 1 |
| PC aa C40:5 | 0.1 | 1 |  | 0.073 | 1 |  | 0.044 | 1 |  | 0.0057 | 1 |  | 0.14 | 1 |
| PC aa C40:6 | -0.0076 | 1 |  | 0.023 | 1 |  | -0.0078 | 1 |  | 0.039 | 1 |  | 0.045 | 1 |
| PC aa C42:0 | -0.0026 | 1 |  | -0.0068 | 1 |  | -0.035 | 1 |  | 0.079 | 1 |  | 0.15 | 1 |
| PC aa C42:1 | -0.01 | 1 |  | -0.037 | 1 |  | -0.021 | 1 |  | 0.067 | 1 |  | 0.058 | 1 |
| PC aa C42:2 | 0.05 | 1 |  | 0.044 | 1 |  | 0.038 | 1 |  | 0.068 | 1 |  | 0.064 | 1 |
| PC aa C42:4 | -0.059 | 1 |  | -0.027 | 1 |  | -0.044 | 1 |  | -0.0087 | 1 |  | 0.11 | 1 |
| PC aa C42:5 | 0.017 | 1 |  | -0.012 | 1 |  | -0.018 | 1 |  | -0.026 | 1 |  | 0.011 | 1 |
| PC aa C42:6 | 0.039 | 1 |  | -0.0024 | 1 |  | 0.026 | 1 |  | -0.026 | 1 |  | -0.035 | 1 |
| PC aa C43:6 | 0.0082 | 1 |  | -0.049 | 1 |  | -0.011 | 1 |  | -0.026 | 1 |  | 0.046 | 1 |
| PC ae C30:0 | 0.073 | 1 |  | 0.0022 | 1 |  | 0.061 | 1 |  | 0.092 | 1 |  | 0.028 | 1 |
| PC ae C32:0 | 0.072 | 1 |  | 0.006 | 1 |  | 0.0066 | 1 |  | 0.021 | 1 |  | 0.065 | 1 |
| PC ae C32:1 | 0.042 | 1 |  | -0.021 | 1 |  | -0.0028 | 1 |  | 0.0011 | 1 |  | 0.057 | 1 |
| PC ae C32:2 | 0.028 | 1 |  | -0.025 | 1 |  | -0.021 | 1 |  | 0.015 | 1 |  | 0.099 | 1 |
| PC ae C34:0 | 0.082 | 1 |  | 0.033 | 1 |  | 0.019 | 1 |  | 0.009 | 1 |  | 0.074 | 1 |
| PC ae C34:1 | 0.066 | 1 |  | -0.0037 | 1 |  | 0.00038 | 1 |  | -0.062 | 1 |  | 0.11 | 1 |
| PC ae C34:2 | 0.076 | 1 |  | -0.069 | 1 |  | -0.0099 | 1 |  | -0.014 | 1 |  | 0.16 | 1 |
| PC ae C34:3 | 0.038 | 1 |  | -0.11 | 0.81 |  | -0.029 | 1 |  | -0.065 | 1 |  | 0.062 | 1 |
| PC ae C34:4 | 0.085 | 1 |  | 0.0077 | 1 |  | 0.0091 | 1 |  | 0.034 | 1 |  | 0.051 | 1 |
| PC ae C36:0 | 0.05 | 1 |  | -0.0038 | 1 |  | 0.0068 | 1 |  | 0.013 | 1 |  | 0.037 | 1 |
| PC ae C36:1 | 0.054 | 1 |  | 0.006 | 1 |  | 0.0062 | 1 |  | -0.019 | 1 |  | 0.084 | 1 |
| PC ae C36:2 | 0.078 | 1 |  | -0.0092 | 1 |  | -0.0024 | 1 |  | 0.069 | 1 |  | 0.19 | 1 |
| PC ae C36:3 | 0.07 | 1 |  | -0.059 | 1 |  | -0.038 | 1 |  | -0.017 | 1 |  | 0.19 | 1 |
| PC ae C36:4 | 0.055 | 1 |  | -0.05 | 1 |  | -0.035 | 1 |  | -0.023 | 1 |  | 0.17 | 1 |
| PC ae C36:5 | 0.041 | 1 |  | -0.055 | 1 |  | -0.031 | 1 |  | -0.087 | 1 |  | 0.062 | 1 |
| PC ae C36:6 | 0.098 | 1 |  | 0.035 | 1 |  | 0.035 | 1 |  | -0.02 | 1 |  | 0.029 | 1 |
| PC ae C38:0 | 0.013 | 1 |  | 0.015 | 1 |  | -0.013 | 1 |  | 0.086 | 1 |  | 0.086 | 1 |
| PC ae C38:2 | 0.13 | 0.45 |  | -0.021 | 1 |  | 0.063 | 1 |  | 0.0059 | 1 |  | 0.14 | 1 |
| PC ae C38:3 | 0.084 | 1 |  | -0.004 | 1 |  | 0.011 | 1 |  | 0.024 | 1 |  | 0.18 | 1 |
| PC ae C38:4 | 0.057 | 1 |  | -0.042 | 1 |  | -0.017 | 1 |  | -0.0024 | 1 |  | 0.2 | 1 |
| PC ae C38:5 | 0.037 | 1 |  | -0.052 | 1 |  | -0.045 | 1 |  | -0.058 | 1 |  | 0.14 | 1 |
| PC ae C38:6 | 0.026 | 1 |  | -0.041 | 1 |  | -0.051 | 1 |  | 0.055 | 1 |  | 0.14 | 1 |
| PC ae C40:0 | -0.029 | 1 |  | -0.021 | 1 |  | -0.034 | 1 |  | -0.055 | 1 |  | -0.039 | 1 |
| PC ae C40:1 | 0.021 | 1 |  | -0.011 | 1 |  | -0.0076 | 1 |  | -0.039 | 1 |  | 0.039 | 1 |
| PC ae C40:2 | 0.098 | 1 |  | 0.052 | 1 |  | 0.013 | 1 |  | 0.12 | 1 |  | 0.17 | 1 |
| PC ae C40:3 | 0.013 | 1 |  | -0.055 | 1 |  | -0.022 | 1 |  | 0.000098 | 1 |  | 0.16 | 1 |
| PC ae C40:4 | 0.065 | 1 |  | -0.048 | 1 |  | 0.0031 | 1 |  | -0.014 | 1 |  | 0.17 | 1 |
| PC ae C40:5 | 0.02 | 1 |  | -0.055 | 1 |  | -0.056 | 1 |  | 0.0041 | 1 |  | 0.18 | 1 |
| PC ae C40:6 | 0.022 | 1 |  | -0.028 | 1 |  | -0.027 | 1 |  | 0.15 | 1 |  | 0.19 | 1 |
| PC ae C42:0 | 0.0011 | 1 |  | -0.0023 | 1 |  | -0.0067 | 1 |  | 0.053 | 1 |  | 0.025 | 1 |
| PC ae C42:1 | 0.027 | 1 |  | -0.0059 | 1 |  | 0.014 | 1 |  | -0.061 | 1 |  | 0.005 | 1 |
| PC ae C42:2 | 0.081 | 1 |  | 0.011 | 1 |  | 0.047 | 1 |  | -0.0028 | 1 |  | 0.019 | 1 |
| PC ae C42:3 | 0.047 | 1 |  | -0.021 | 1 |  | -0.0047 | 1 |  | 0.08 | 1 |  | 0.099 | 1 |
| PC ae C42:4 | 0.034 | 1 |  | -0.054 | 1 |  | -0.00044 | 1 |  | -0.08 | 1 |  | 0.074 | 1 |
| PC ae C42:5 | 0.037 | 1 |  | -0.018 | 1 |  | -0.0092 | 1 |  | 0.01 | 1 |  | 0.14 | 1 |
| PC ae C42:6 | 0.0076 | 1 |  | -0.044 | 1 |  | -0.024 | 1 |  | 0.0085 | 1 |  | 0.095 | 1 |
| SM C35:0 | 0.037 | 1 |  | 0.0036 | 1 |  | -0.0022 | 1 |  | -0.00094 | 1 |  | 0.087 | 1 |
| SM C35:1 | 0.035 | 1 |  | -0.018 | 1 |  | -0.019 | 1 |  | 0.09 | 1 |  | 0.1 | 1 |
| SM C36:0 | 0.007 | 1 |  | 0.025 | 1 |  | 0.018 | 1 |  | -0.075 | 1 |  | 0.028 | 1 |
| SM C36:1 | 0.035 | 1 |  | -0.011 | 1 |  | -0.0076 | 1 |  | 0.021 | 1 |  | 0.094 | 1 |
| SM C36:2 | 0.025 | 1 |  | -0.036 | 1 |  | -0.027 | 1 |  | 0.078 | 1 |  | 0.16 | 1 |
| SM C37:1 | 0.092 | 1 |  | 0.047 | 1 |  | 0.024 | 1 |  | 0.14 | 1 |  | 0.11 | 1 |
| SM C38:1 | 0.069 | 1 |  | -0.0073 | 1 |  | 0.016 | 1 |  | -0.021 | 1 |  | 0.21 | 1 |
| SM C38:2 | 0.042 | 1 |  | -0.011 | 1 |  | 0.0011 | 1 |  | 0.03 | 1 |  | 0.13 | 1 |
| SM C38:3 | 0.055 | 1 |  | 0.046 | 1 |  | 0.044 | 1 |  | 0.029 | 1 |  | 0.033 | 1 |
| SM C39:1 | 0.079 | 1 |  | 0.0021 | 1 |  | -0.0011 | 1 |  | 0.15 | 1 |  | 0.16 | 1 |
| SM C39:2 | 0.064 | 1 |  | -0.022 | 1 |  | 0.038 | 1 |  | 0.077 | 1 |  | 0.066 | 1 |
| SM C40:1 | 0.05 | 1 |  | -0.0034 | 1 |  | -0.0014 | 1 |  | 0.034 | 1 |  | 0.15 | 1 |
| SM C40:2 | 0.051 | 1 |  | -0.033 | 1 |  | -0.015 | 1 |  | 0.012 | 1 |  | 0.16 | 1 |
| SM C40:3 | 0.05 | 1 |  | 0.036 | 1 |  | 0.067 | 1 |  | 0.025 | 1 |  | 0.0011 | 1 |
| SM C40:4 | 0.031 | 1 |  | -0.0021 | 1 |  | -0.0041 | 1 |  | -0.063 | 1 |  | 0.099 | 1 |
| SM C39:5 | 0.028 | 1 |  | -0.037 | 1 |  | -0.034 | 1 |  | 0.0013 | 1 |  | 0.051 | 1 |
| SM C41:1 | 0.076 | 1 |  | 0.023 | 1 |  | 0.014 | 1 |  | 0.11 | 1 |  | 0.17 | 1 |
| SM C41:2 | 0.089 | 1 |  | 0.01 | 1 |  | 0.025 | 1 |  | 0.2 | 1 |  | 0.19 | 1 |
| SM C42:1 | 0.076 | 1 |  | 0.027 | 1 |  | 0.047 | 1 |  | 0.035 | 1 |  | 0.12 | 1 |
| SM C42:2 | 0.02 | 1 |  | -0.015 | 1 |  | -0.026 | 1 |  | -0.0096 | 1 |  | 0.088 | 1 |
| SM C42:3 | -0.014 | 1 |  | -0.057 | 1 |  | -0.068 | 1 |  | 0.026 | 1 |  | 0.1 | 1 |
| SM C41:3 | 0.0068 | 1 |  | -0.048 | 1 |  | -0.031 | 1 |  | 0.011 | 1 |  | 0.0086 | 1 |
| SM C42:4 | 0.026 | 1 |  | -0.01 | 1 |  | -0.042 | 1 |  | -0.031 | 1 |  | 0.22 | 1 |
| SM C42:6 | -0.043 | 1 |  | -0.035 | 1 |  | -0.049 | 1 |  | -0.026 | 1 |  | 0.04 | 1 |
| SM C43:1 | 0.03 | 1 |  | -0.0023 | 1 |  | -0.015 | 1 |  | 0.053 | 1 |  | 0.12 | 1 |
| SM C43:2 | 0.031 | 1 |  | 0.009 | 1 |  | -0.0018 | 1 |  | 0.077 | 1 |  | 0.053 | 1 |
| SM C43:0 | 0.05 | 1 |  | 0.0022 | 1 |  | 0.019 | 1 |  | 0.14 | 1 |  | 0.1 | 1 |
| SM C44:2 | 0.073 | 1 |  | 0.032 | 1 |  | 0.045 | 1 |  | -0.005 | 1 |  | 0.078 | 1 |
| SM C43:3 | 0.02 | 1 |  | -0.031 | 1 |  | -0.027 | 1 |  | 0.053 | 1 |  | 0.044 | 1 |
| SM C44:6 | 0.039 | 1 |  | 0.014 | 1 |  | 0.033 | 1 |  | 0.039 | 1 |  | 0.051 | 1 |
| NEFA 10:0 | 0.093 | 1 |  | -0.0057 | 1 |  | 0.04 | 1 |  | -0.019 | 1 |  | 0.083 | 1 |
| NEFA 12:0 | 0.11 | 0.94 |  | 0.03 | 1 |  | 0.098 | 1 |  | 0.054 | 1 |  | -0.013 | 1 |
| NEFA 12:1 | 0.053 | 1 |  | 0.027 | 1 |  | 0.036 | 1 |  | 0.038 | 1 |  | 0.027 | 1 |
| NEFA 14:0 | 0.094 | 1 |  | 0.027 | 1 |  | 0.059 | 1 |  | 0.096 | 1 |  | 0.032 | 1 |
| NEFA 14:1 | 0.088 | 1 |  | 0.049 | 1 |  | 0.063 | 1 |  | 0.047 | 1 |  | -0.0039 | 1 |
| NEFA 15:0 | 0.076 | 1 |  | 0.038 | 1 |  | 0.038 | 1 |  | 0.044 | 1 |  | 0.058 | 1 |
| NEFA 15:1 | 0.091 | 1 |  | 0.096 | 1 |  | 0.055 | 1 |  | 0.11 | 1 |  | 0.022 | 1 |
| NEFA 16:0 | 0.065 | 1 |  | -0.00094 | 1 |  | 0.032 | 1 |  | 0.038 | 1 |  | -0.012 | 1 |
| NEFA 16:1 | 0.06 | 1 |  | 0.047 | 1 |  | 0.05 | 1 |  | 0.014 | 1 |  | -0.05 | 1 |
| NEFA 16:2 | 0.081 | 1 |  | 0.013 | 1 |  | 0.039 | 1 |  | -0.0041 | 1 |  | 0.041 | 1 |
| NEFA 17:0 | 0.068 | 1 |  | 0.0077 | 1 |  | 0.029 | 1 |  | -0.0064 | 1 |  | 0.0096 | 1 |
| NEFA 17:1 | 0.064 | 1 |  | 0.032 | 1 |  | 0.035 | 1 |  | -0.025 | 1 |  | -0.0096 | 1 |
| NEFA 18:0 | 0.057 | 1 |  | -0.028 | 1 |  | 0.034 | 1 |  | -0.041 | 1 |  | -0.04 | 1 |
| NEFA 18:1 | 0.055 | 1 |  | 0.0077 | 1 |  | 0.023 | 1 |  | 0.017 | 1 |  | -0.016 | 1 |
| NEFA 18:2 | 0.049 | 1 |  | -0.0079 | 1 |  | 0.017 | 1 |  | 0.056 | 1 |  | 0.014 | 1 |
| NEFA 18:3 | 0.08 | 1 |  | 0.011 | 1 |  | 0.028 | 1 |  | -0.007 | 1 |  | 0.00068 | 1 |
| NEFA 19:1 | 0.069 | 1 |  | 0.029 | 1 |  | 0.038 | 1 |  | 0.014 | 1 |  | -0.022 | 1 |
| NEFA 20:0 | 0.068 | 1 |  | 0.014 | 1 |  | 0.051 | 1 |  | -0.13 | 1 |  | -0.089 | 1 |
| NEFA 20:2 | 0.014 | 1 |  | -0.014 | 1 |  | 0.0088 | 1 |  | -0.033 | 1 |  | -0.081 | 1 |
| NEFA 20:3 | 0.025 | 1 |  | 0.03 | 1 |  | 0.02 | 1 |  | -0.042 | 1 |  | -0.15 | 1 |
| NEFA 20:4 | 0.034 | 1 |  | 0.026 | 1 |  | 0.021 | 1 |  | -0.069 | 1 |  | -0.058 | 1 |
| NEFA 20:5 | 0.078 | 1 |  | -0.01 | 1 |  | 0.035 | 1 |  | 0.023 | 1 |  | -0.12 | 1 |
| NEFA 22:4 | 0.045 | 1 |  | 0.00058 | 1 |  | 0.019 | 1 |  | -0.08 | 1 |  | -0.055 | 1 |
| NEFA 22:5 | 0.045 | 1 |  | 0.013 | 1 |  | 0.02 | 1 |  | -0.0064 | 1 |  | -0.024 | 1 |
| NEFA 22:6 | 0.0023 | 1 |  | -0.0022 | 1 |  | -0.0034 | 1 |  | 0.012 | 1 |  | -0.066 | 1 |
| NEFA 24:0 | 0.072 | 1 |  | 0.085 | 1 |  | 0.078 | 1 |  | -0.09 | 1 |  | -0.038 | 1 |
| NEFA 24:2 | 0.017 | 1 |  | -0.021 | 1 |  | -0.013 | 1 |  | 0.045 | 1 |  | 0.038 | 1 |
| NEFA 24:4 | -0.014 | 1 |  | -0.073 | 1 |  | -0.017 | 1 |  | -0.15 | 1 |  | -0.087 | 1 |
| NEFA 24:5 | 0.0095 | 1 |  | -0.031 | 1 |  | 0.0094 | 1 |  | -0.14 | 1 |  | -0.013 | 1 |
| NEFA 26:1 | 0.0054 | 1 |  | -0.0071 | 1 |  | -0.025 | 1 |  | -0.044 | 1 |  | 0.033 | 1 |
| Sum NEFA | 0.073 | 1 |  | -0.0035 | 1 |  | 0.032 | 1 |  | 0.044 | 1 |  | -0.017 | 1 |

Abbreviations: Hpro , Hydroxy-Proline, Carn, acylcarnitine; LCA, long-chain acylcarnitines; LPC, lysophosphatidylcholine; PCaa, diacyl-phosphatidylcholine; PCae, acyl-alkyl-phosphatidylcholine; SM, sphingomyeline; NEFA, non-esterified acids
